# Swelling‐Dependent Shape‐Based Transformation of a Human Mesenchymal Stromal Cells‐Laden 4D Bioprinted Construct for Cartilage Tissue Engineering

**DOI:** 10.1002/adhm.202201891

**Published:** 2022-11-17

**Authors:** Pedro J. Díaz‐Payno, Maria Kalogeropoulou, Iain Muntz, Esther Kingma, Nicole Kops, Matteo D'Este, Gijsje H. Koenderink, Lidy E. Fratila‐Apachitei, Gerjo J. V. M. van Osch, Amir A. Zadpoor

**Affiliations:** ^1^ Department of Biomechanical Engineering Faculty of Mechanical Maritime and Materials Engineering Delft University of Technology Delft 2628CD Netherlands; ^2^ Department of Orthopedics and Sports Medicine Erasmus MC University Medical Center Rotterdam 3015GD Netherlands; ^3^ Department of Bionanoscience Kavli Institute of Nanoscience Delft Delft University of Technology Delft 2628CD Netherlands; ^4^ AO Research Institute Davos Davos 7270 Switzerland; ^5^ Department of Otorhinolaryngology Erasmus MC University Medical Center Rotterdam 3015GD Netherlands

**Keywords:** 4D bioprinting, biofabrication, shape‐change, smart bioinks, tissue engineering

## Abstract

3D bioprinting is usually implemented on flat surfaces, posing serious limitations in the fabrication of multilayered curved constructs. 4D bioprinting, combining 3D bioprinting with time‐dependent stimuli‐induced transformation, enables the fabrication of shape‐changing constructs. Here, a 4D biofabrication method is reported for cartilage engineering based on the differential swelling of a smart multi‐material system made from two hydrogel‐based materials: hyaluronan and alginate. Two ink formulations are used: tyramine‐functionalized hyaluronan (HAT, high‐swelling) and alginate with HAT (AHAT, low‐swelling). Both inks have similar elastic, shear‐thinning, and printability behavior. The inks are 3D printed into a bilayered scaffold before triggering the shape‐change by using liquid immersion as stimulus. In time (4D), the differential swelling between the two zones leads to the scaffold's self‐bending. Different designs are made to tune the radius of curvature and shape. A bioprinted formulation of AHAT and human bone marrow cells demonstrates high cell viability. After 28 days in chondrogenic medium, the curvature is clearly present while cartilage‐like matrix production is visible on histology. A proof‐of‐concept of the recently emerged technology of 4D bioprinting with a specific application for the design of curved structures potentially mimicking the curvature and multilayer cellular nature of native cartilage is demonstrated.

## Graphical abstract

3D bioprinting poses serious limitations in the fabrication of multilayered curved constructs, motivating the development of 4D bioprinting as the next generation of biofabrication technologies. 4D bioprinting, combining 3D bioprinting with time‐dependent stimuli‐induced transformation, enables the fabrication of self‐bending constructs. Here, a 4D smart multi‐material system for curved cartilage engineering is reported as a proof‐of‐concept.

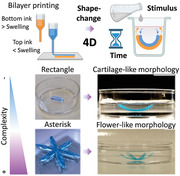

## 1 Introduction

3D bioprinting is a powerful and versatile technology for the fabrication of biomimetic tissue constructs, enabling an unprecedented level of control over the composition and geometry of the printed structures. This technology supports the fabrication of highly engineered scaffolds, capable of closely recapitulating the heterogeneity and complexity of native tissues.^[^ ^1^, ^2^, ^3^ ^]^ In 3D bioprinting, cell‐laden hydrogels, collectively known as “bioinks”, are used for the fabrication of living scaffolds in a layer‐by‐layer manner.^[^ ^4^ ^]^ There are several bioprinting techniques that can be categorized as follows: i) extrusion‐based, including mechanical or pneumatic;^[^ ^5^ ^]^ ii) jetting‐based, including inkjet, microvalve, laser‐assisted or acoustic;^[^ ^6^ ^]^ and iii) vat polymerization‐based, including stereolithography, digital light processing or two‐photon polymerization.^[^ ^7^ ^]^ Among these different approaches, particular focus has been placed on extrusion‐based bioprinting,^[^ ^8^, ^9^ ^]^ due to its distinct advantages including multi‐head systems for simultaneous use of one or more biomaterials.^[^ ^10^ ^]^ During extrusion‐based bioprinting, a bioink is pushed through a nozzle and is deposited on the printing platform using a predetermined printing pattern, in successive 2D layers, until the 3D scaffold is fabricated. While 3D bioprinting appeared as a promising tissue engineering (TE) approach, it has significant limitations in the engineering of complex out‐of‐plane features and shapes, and temporally varying multi‐material constructs.^[^ ^11^ ^]^ These limitations are particularly evident in the fabrication of tubular or curved living structures and usually require the use of sacrificial materials and supports, introducing additional post‐printing processing steps and, thus, increasing the total fabrication time.^[^ ^11^, ^12^ ^]^ For instance, standard 3D bio/printing would need a significant number of print‐head changes, involving complex coding, to be able to fabricate both a porous multi‐layered and curved scaffold. Moreover, optimal tissue formation often requires temporal changes in the geometry and properties of the biomaterial. These spatial and temporal complexities can be best addressed with 4D (4D = 3D + time) bioprinting as the next generation of biofabrication technologies.

4D bioprinting uses the same fabrication principles as 3D bioprinting whilst introducing a time‐dependent post‐printing transformation during which the application of one or more stimuli triggers the transformation of the printed structure, introducing time as the 4D.^[^ ^13^, ^14^, ^15^ ^]^ 4D bioprinting enables the fabrication of more sophisticated configurations, which may better mimic tissue complexity (e.g., organ shape, multilayer nature), as well as the capability to reshape the structure of a construct over time for practical needs (e.g., minimally invasive scaffolds). The most commonly used transformation in 4D printing is based on shape‐change, and can be used for the fabrication of self‐bending constructs.^[^ ^16^ ^]^ The applied stimuli can be a temperature change,^[^ ^17^ ^]^ immersion in aqueous solutions,^[^ ^18^ ^]^ electric potential,^[^ ^19^ ^]^ or magnetic stimulation,^[^ ^20^ ^]^ as well as light irradiation.^[^ ^21^, ^22^, ^23^ ^]^ Though still in its infancy, 4D bioprinting has already been used in a few studies, yielding promising results and potential applications in tissues such as cartilage,^[^ ^22^, ^23^, ^24^ ^]^ trachea,^[^ ^18^ ^]^ muscle,^[^ ^19^ ^]^ kidney,^[^ ^21^ ^]^ or vascular/neural network.^[^ ^25^ ^]^ However, most of these studies fail to tackle the fundamental questions regarding the relation between the printing design and the limitations of the 4D bioprinting systems to build complex structures. These questions need to be answered to better understand the capabilities of this new technology. In addition, some of these studies present limitations regarding the relatively short‐term in vitro evaluation (a few days), therefore lacking information on whether the shape‐change is stable over time, or on what are the long‐term effects of shape transformation on cell differentiation and cell‐derived matrix deposition. Finally, some of the fabrication strategies or stimuli used (heat, UV) can cause potential cell damage, which is often overlooked.^[^ ^22^, ^23^, ^26^, ^27^, ^28^ ^]^

Nature‐derived hydrogels are often chosen as cell carriers due to their cytocompatibility, water storage or swelling capacity, and their ability to support tissue deposition. It is, therefore, key to find 4D strategies that are compatible with the current hydrogel‐based bioink systems. Out‐of‐plane transformations (i.e., 2D to 3D shape‐shifting) can be created through the differential growth of a bilayer made from two materials with different stimulus‐response behaviors.^[^ ^29^, ^30^ ^]^ Various classes of hydrogels have inherently different swelling behaviors, which can be harnessed for 4D bioprinting. Recently, the use of a bioink made of hyaluronan functionalized with a tyramine‐group (HAT) was proposed as a promising biomaterial for TE,^[^ ^31^ ^]^ and especially for cartilage TE.^[^ ^32^, ^33^, ^34^, ^35^ ^]^ Alginate is another natural hydrogel which is extensively used in bioprinting in general^[^ ^36^, ^37^, ^38^, ^39^, ^40^, ^41^ ^]^ and in cartilage engineering in particular.^[^ ^39^, ^42^, ^43^, ^44^ ^]^ That is because it is non‐toxic,^[^ ^45^, ^46^ ^]^ low‐immunogenic,^[^ ^47^ ^]^ and can gelate into a 3D structure upon exposure to Ca^2+^ ions.^[^ ^48^, ^49^ ^]^

Here, we report an advanced 4D biofabrication method based on the differential swelling of a multi‐material smart hydrogel‐based bioink, with a special focus on cartilage TE. This approach allows the fabrication of bilayered scaffolds made from a bottom part of a HAT precursor and a composite hydrogel top part made of alginate mixed with HAT (AHAT), capable of self‐bending upon immersion in aqueous solutions due to swelling‐based differential material growth. The mechanism behind this growth is the volume increase of the material due to water absorbtion capacity, which is different in each material and triggers the bilayer to curve. The degree of obtained curvature was adjusted by tuning a range of parameters including the infill density and printing angle, the thickness of each layer, the CaCl_2_ crosslinking time, as well as the type of the swelling solvent. The incorporation of human bone marrow‐derived stromal/stem cells (hMSCs) in the composite hydrogel allowed for the fabrication of living, self‐bending scaffolds that could support high cell viability and cartilage‐like tissue deposition after 28 days in culture. Hence, this is one of the very few studies considering 4D bioprinting and the implications of the design in the final curvature to report the incorporation of human‐derived cells in a self‐bending bilayered scaffold made from two commonly used natural hydrogels in cartilage TE and cultured for such a duration in vitro.

## 2 Results

**Figure 1:**
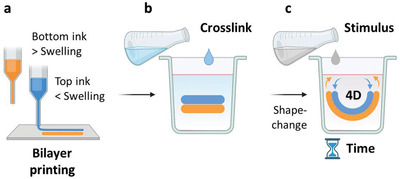
4D printing concept based on swelling‐based differential growth. a) A schematic drawing of the two inks 3D printed into a bilayer: high swelling (bottom layer, in orange) and low swelling (top layer, in blue). b) The scaffold is then crosslinked to obtain a solid construct. c) Finally, a swelling‐based stimulus is applied on the construct, which triggers the construct's shape‐based transformation behavior over time (4D).

The basic concept of this study is a 4D printing approach based on the differential volumetric swelling creating a differential growth of a bilayer printed with two different inks (**Figure** **1**). For the bottom layer, a highly swellable HAT was selected as ink. For the top layer, the AHAT composite with lower swelling compared with bottom layer was selected. Once printed into a bilayer, the scaffold was ionically crosslinked to form a solid construct, which was then allowed to swell (stimulus) for a certain period of time. These materials had different swelling ratios after being ionically crosslinked, which caused the differential growth and self‐bending of the scaffolds.

### 2.1 Rheological Characterization of the Novel Inks Demonstrates Suitability for Extrusion‐Based 3D Printing

**Figure 2:**
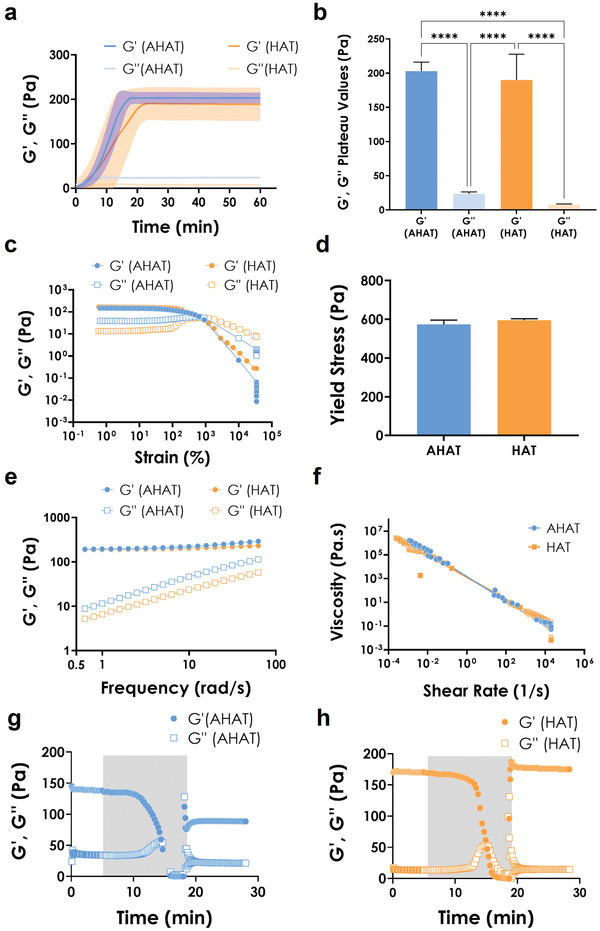
The rheological characterization of the inks made of Alginate/Hyaluronan‐ Tyramine (AHAT) and Hyaluronan‐Tyramine (HAT) for extrusion‐based 3D printing. a) The time dependence of the linear storage (G’) and loss modulus (G”) of AHAT (blue) and HAT (orange). The shaded area represents the standard deviation. b) The plateau values of G’ and G” for AHAT (blue) and HAT (orange). c) The amplitude strain sweep for the AHAT (blue) and HAT (orange) inks. The storage moduli, G’, are plotted as circles while loss moduli, G”, are plotted as squares. d) The yield stress values of AHAT (blue) and HAT (orange) determined by large amplitude oscillatory shear. e) The frequency dependence of the storage (G’) (circles) and loss moduli (G”) (squares) of the AHAT (blue) and HAT (orange) inks. f) The shear rate, *γ*˙, dependence of the shear viscosity, *η*, of the AHAT (blue) and HAT (orange). g) The oscillatory thixotropy tests for AHAT and h) HAT, where the shaded area represents the second step in which the stress was increased from 1 Pa to 5 kPa logarithmically, while the non‐shaded area was kept at a constant strain of 0.5% (Figure S1, Supporting Information).

Rheological properties are key to extrusion printing, therefore, we first characterized the relevant rheological features of the new ink and compared them to those of HAT ink.^[^ ^50^ ^]^ Immediately after pre‐crosslinking, the time‐progress of the pre‐crosslinking process to produce the shear‐thinning inks was monitored. The storage or elastic moduli (*G'*) of both inks showed an almost linear, sharp increase before reaching a plateau value at ≈ 20 min, which indicated the end of crosslinking (**Figure** **2**a). A similar behavior, though less pronounced due to the magnitude difference, was also observed for the loss or viscous moduli (*G“*) of both inks. The prevalence of the elastic over the viscous component was observed in both inks, as their *G'* plateau values (*G'*_(AHAT)_ = 203 Pa; *G'*_(HAT)_ = 190 Pa) were at least 8 times greater than their corresponding *G”* values (G“_(AHAT)_ = 23.6 Pa; *G”*_(HAT)_ = 7.9 Pa) (Figure 2b). The calculated damping factor (tan *δ* = *G“/G'*; **Table** **1**) was higher for the AHAT ink than for the HAT ink, revealing the presence of a higher viscous component in the AHAT ink (tan *δ*_(AHAT)_ = 0.116 vs tan *δ*_(HAT)_ = 0.041). Nevertheless, the tan *δ* of either ink was below unity, indicating that the inks are in a gel state.^[^ ^43^ ^]^ The strain sweep tests confirmed an elastic, gel‐like behavior for both inks (Figure 2c), as the *G'* was higher than the *G”* in the linear viscoelastic regions of the *G'*(*γ*) and *G“*(*γ*) curves (where *G'* and *G”* values are independent of the applied deformation). In addition, it was observed that both inks had a similar yield strain (≈ 10^3^%) and yield stress point (≈ 600 Pa; Figure 2d). The frequency dependence of the viscoelastic behavior of the inks is illustrated in Figure 2e. The *G'* values were consistently higher than the *G"* values, showing that the elastic behavior of the two inks was dominant (Figure 2e). The storage moduli of both inks showed a slight frequency dependence, while the loss moduli showed a stronger frequency dependence. This suggested that, although the inks demonstrated a gel‐like behavior,^[^ ^59^ ^]^ the viscous component of the inks has a major influence on the material's behavior when the inks are exposed to higher frequency deformations. The viscosity curve (steady flow tests) revealed that the viscosity of both inks displayed a pronounced shear rate (*γ*) dependence, indicating shear thinning behavior (Figure 2f). Based on the value of the material parameter, *n*, which was determined using the Ostwald‐de Waele power law equation,^[^ ^51^ ^]^ the inks were confirmed to have a shear‐thinning behavior (*n* < 1, Table 1). To allow layer‐by‐layer 3D printing, the materials should be capable of quickly recovering their elastic behavior after extrusion. To test this, we performed a thixotropy test where a constant strain of 0.5% was kept for the first and last step; while for the second step, the stress was increased from 1 Pa to 5 kPa logarithmically in 51 steps (Figure S1, Supporting Information). In both inks, the storage modulus *G'* prevailed during the low strain phase, while the loss modulus *G"* dominated in the high strain phase, as the material starts to flow. The response of both inks to the final, low‐strain step, demonstrated a fast structural recovery, reaching 100% in the case of HAT within 10 min. For AHAT, the storage modulus *G'* reached ≈ 65% of its initial value (Figure 2g). For HAT, the storage modulus *G'* recovered ≈ 100% of the initial value (Figure 2h).

**Table 1:** The rheological properties of Alginate+Hyaluronan‐Tyramine (AHAT) and Hyaluronan‐Tyramine (HAT) inks (n = 6) performed at 21 °C

Ink	Storage Modulus [*G’*]	Loss Modulus [*G’’*]	tan *δ*	Yield Stress [Pa]	*K* [Pa s^n^]	*n*	*R* ^2^
AHAT (1% A; 2.5% HAT)	203 ± 13	23.6 ± 3	0.116	574 ± 22	2467	0.2683	0.9995
HAT (2.5%)	190 ± 38	7.9 ± 1	0.041	595 ± 7	2351	0.1541	0.9996

### 2.2 3D Printed AHAT Scaffolds are Stiffer and Swell less than HAT Scaffolds after Ionic Crosslinking

**Figure 3:**
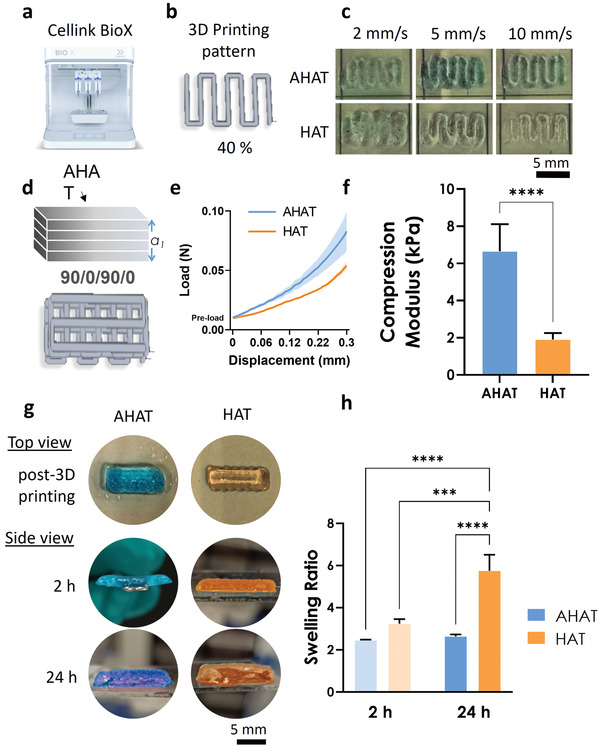
The characterization of the stiffness and swelling ratio of the 3D printed scaffolds after ionic crosslinking. a) The BioX Cellink printer used for the studies (reproduced with Cellink permission). b) The pattern design of one layer (40% infill rectangle) used to optimize the printing parameters of the biomaterial inks. c) Representative images of one layer printed at different speeds (i.e., 2, 5, and 10 mm s^−1^) using the design in (b) for both inks. d) Top: Schematic drawing of the 3D scaffolds containing 4 layers fabricated with either the AHAT or HAT ink and the printing pattern design of the scaffolds organized in the raster angles of 90/0/90/0 degrees for each layer. e) The load versus displacement curve of the 3D scaffolds under unconfined compression test. The standard deviation is depicted using a shadowed color (both groups, n = 3). f) The Young's modulus of the 3D scaffolds obtained from the strain versus stress curve (n = 3). g) Representative images of the ionically crosslinked 3D printed scaffolds using the designs in (d), for each ink: either AHAT (blue) or HAT (orange) at different time‐points: post‐fabrication, 2 and 24 h after immersion in 0.9% NaCl. h) The swelling ratio of each ionically crosslinked 3D printed type of scaffold after 2 and 24 h of immersion (n = 3).

After rheological analyses, the inks were used in the bioprinter (**Figure** **3**a). The printability of the inks was tested by printing a rectilinear pattern (Figure 3b). The printing optimization was performed at different speeds while keeping a constant pressure of ≈45 kPa. For the used pressure, a print head speed of 10 mm s^−1^ was determined to be the best writing speed, leading to an apparent higher fidelity‐ratio for both inks (Figure 3c). Single‐material rectangular 3D constructs of 4 layers (Figure 3d) made from the AHAT or HAT inks were printed to measure their individual responses under compressive loading as well as their swelling behaviors. Based on previous work,^[^ ^39^ ^]^ a rectilinear pattern obtained by varying the degree of patterning 90 degrees from layer to layer was chosen to print the scaffolds (Figure 3d).

The post‐printing ionic crosslinking of the AHAT construct with 200 mm CaCl_2_ for 10 min resulted in a threefold increase in the compressive modulus (≈6.7 kPa) relative to the HAT scaffold as determined by unconfined compression (Figure 3e,f). Moreover, the AHAT constructs swelled less than the HAT constructs after 24 h (Figure 3g,h). After 2 h of immersion in a saline medium containing 0.9% NaCl and 2 mm CaCl_2_, the swelling ratio of AHAT was ≈2.5, while it was 2.6 after 24 h. On the other hand, the HAT scaffolds exhibited a pronounced increase in the swelling ratio from ≈3.2 measured after 2 h of immersion to ≈5.7 after 24 h, indicating that the HAT scaffolds absorbed at least two times more liquid than their AHAT counterparts (within 24 h).

### 2.3 4D Printing Constructs with a Lower Infill Density Results in a Higher Bending Curvature

**Figure 4:**
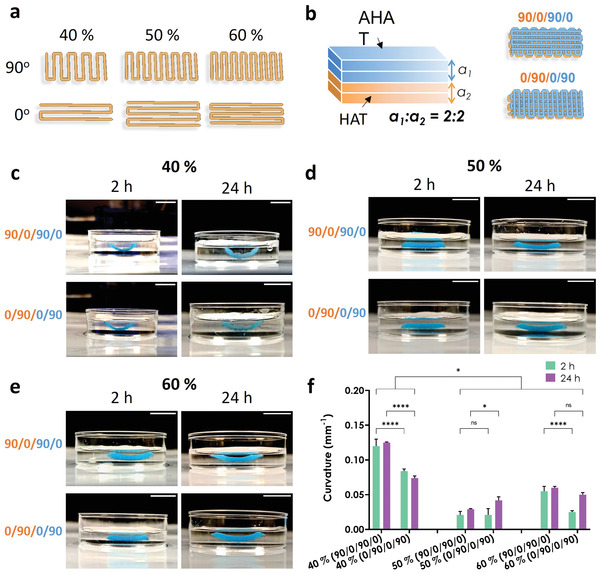
The effects of the printing angle and infill density on the degree of curvature. a) The design of the printing patterns used for the layers with different printing angles (either 90° or 0°) and different infill densities (i.e., 40, 50, or 60%). b) Schematic drawing of the 3D bilayered scaffolds showing the 4 sublayers: the two top sublayers were made of AHAT (blue, a_1_) while the two bottom sublayers were made of HAT (orange, a_2_). The two printing pattern designs of the 3D bilayered scaffolds with different printing angles (90/0/90/0° and 0/90/0/90°) are also presented. c) Representative images of the scaffolds printed with an infill density of 40% and starting either at a printing angle of 90° or at 0°. The photographs were taken either 2 h or 24 h after the application of the stimulus. AHAT is colored in blue, while HAT is translucent (or orange in the schematic drawing). d) Representative images of the scaffolds with an infill density of 50%. e) Representative images of the scaffolds with an infill density of 60%. f) The degree of curvature (1/mm) measured from the pictures of the scaffolds in (c), (d), and (e) (n = 3). The scale bar in all sub‐figures corresponds to a length of 10 mm.

To achieve shape transformation in 4D printing, we used a bilayer made of two materials with differential swelling behavior upon stimulus exposure: a rectangular bilayered scaffold consisting of a top layer of AHAT and a bottom layer of HAT. Since the swelling ratio of AHAT was lower than the swelling ratio of HAT (Figure 3h), the bending of the bilayer upon immersion was expected to be concave upward, towards the top AHAT layer. We varied the infill density and the printing angles of the layers and evaluated the effects of these parameters on the curvature (**Figure** **4**a,b). Figure S2, Supporting Information, presents some further schematics of the scaffold designs. The greatest curvature value (0.125 mm^−1^) was observed for the scaffolds printed with 40% infill and 90/0/90/0 pattern after 24 h of immersion (Figure 4c, top). For the same infill values, a change in the printing direction (0/90/0/90) resulted in a significantly lower curvature of 0.074 mm^−1^ after 24 h (Figure 4c, bottom). The scaffolds with 50% infill density and 90/0/90/0 printing angle had the lowest curvature (i.e., 0.029 mm^−1^) (Figure 4d, top). For the same infill, the 0/90/0/90 pattern led to an increase in the curvature to 0.042 mm^−1^ after 24 h (Figure 4d bottom). For the scaffolds with an infill density of 60%, the 90/0/90/0 pattern led to similar values of curvature for 2 and 24 h (i.e., 0.055 and 0.06 mm^−1^, respectively) (Figure 4e, top). On the other hand, the scaffolds with the 0/90/0/90 pattern took a long time to reach their final curvature of 0.025 and 0.05 mm^−1^ for 2 and 24 h, respectively (Figure 4e, bottom). Although the 90/0/90/0 scaffolds with an infill of 40% had pronounced self‐bending, the scaffolds were less robust in comparison with their 60% counterparts. The latter design was, therefore, selected for further experiments. The infill density had a significant effect on the degree of curvature, while the printing angle effect was more evident in the constructs designed with a lower infill density than those with a higher infill density (Figure 4f). Other infill densities such as 30% and 70% were preliminary tested (data not shown). However, the scaffolds printed with 30% infill were too weak and unstable to be handled for further analysis and the prints with 70% infill density resulted in compromised 3D structures having too much material due to high spread ratio of the printed filaments.

### 2.4 The Ratio of AHAT:HAT in 3D Printed Bilayer Constructs Modulates the Degree of Curvature

**Figure 5:**
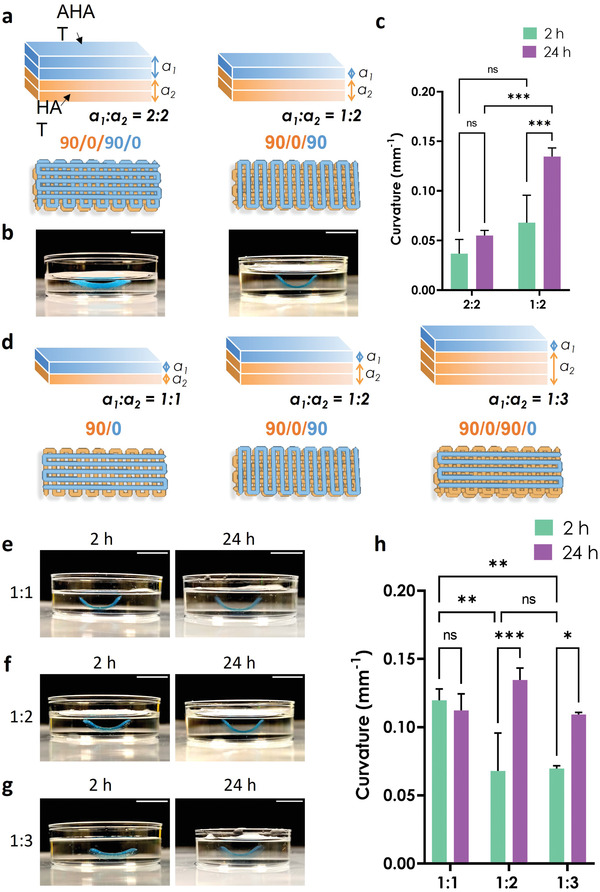
The effects of the bilayer ratio (AHAT:HAT) on the degree of curvature. a) Schematic drawing of the 3D printed biphasic scaffolds made of either 2 layers of each material (left) or 1 layer of AHAT and 2 layers of HAT (right), and the corresponding printing patterns starting with the same printing angle (90°) and infill density (60%). b) Representative images of the scaffolds after 24 h of stimulus application. c) The degree of curvature (1/mm) measured from the pictures of the scaffolds at two different time points (n ≥ 3). d) Schematic drawing of the 3D printed biphasic scaffolds made with three configurations: 1 layer of each material (left), 1 layer of AHAT with 2 layers of HAT (middle), or 1 layer of AHAT and 3 layers of HAT (right). The corresponding printing patterns starting with the same printing angle (90°) and infill density (60%) are also presented. e–g) Representative images of the scaffolds with different AHAT:HAT layer ratios after 2 and 24 h of stimulus application. h) The degree of curvature (1/mm) measured from the photographs of the scaffolds at two different time‐points (n = 3). The scale bars in all the sub‐figures correspond to 10 mm.

Next, we studied whether the ratio of the number of the AHAT layers (low swelling) to that of the HAT (high swelling) layers and the total height of the scaffold had an influence on the degree of curvature achieved by the system. First, the number of the top AHAT layers (thickness) was varied from 2 to 1 layers, while keeping the number of the bottom HAT layers constant at 2 layers (AHAT:HAT ratio 2:2 vs 1:2, **Figure** **5**a). After 2 h of stimulus application, the bilayer scaffolds of both groups had a similar curvature. After 24 h of stimulus application, the bilayer scaffolds with a higher ratio of AHAT:HAT (2:2) exhibited a significantly lower curvature than the scaffolds with a lower ratio (1:2), that is, 0.06 versus 0.13 mm^−1^, respectively (Figure 5b,c). In addition, from 2 to 24 h, only the scaffolds with a lower ratio (1:2) exhibited a significant increase in the curvature of the scaffolds.

Then, we evaluated the influence of varying the number of the bottom HAT layers (1, 2, or 3 layers), while keeping a constant number of 1 top AHAT layer (AHAT:HAT ratio 1:1 vs 1:2 vs 1:3; Figure 5d). 2 h post‐stimulus, the 1:1 scaffolds had a higher curvature (0.12 mm^−1^) than the scaffolds with the ratios of 1:2 (0.068 mm^−1^) and 1:3 (0.07 mm^−1^). After 24 h, however, the curvature of the 1:2 and 1:3 scaffolds had notably increased, reaching 0.13 and 0.11 mm^−1^, respectively, which was similar to the curvature of the 1:1 scaffolds. Although the 1:1 scaffolds showed a fast self‐bending behavior that was evident as early as 2 h post‐immersion, they were very fragile and prone to tearing when manipulated with a spatula. At the other end of the spectrum, the HAT phase of the 1:3 scaffolds swelled substantially, to the extent that it easily separated from the AHAT layer during the scaffold handling as it was “sticking” to the tools. Therefore, the 1:2 thickness ratio was selected for further experiments.

### 2.5 Increasing the Time of the Ionic Crosslinking (200 mm CaCl2) has Limited Effect on the Material Swelling and Stiffness, and on the Curvature of the Bilayer Scaffolds

**Figure 6:**
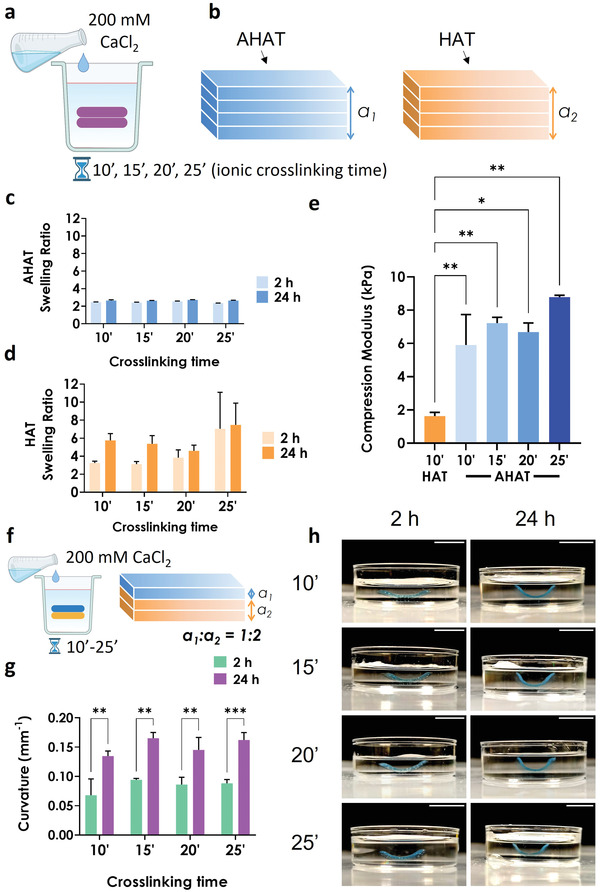
The effects of ionic crosslinking time on the degree of curvature. a) Schematic drawing representing the post‐printing ionic crosslinking treatment with 200 mm CaCl_2_ for 10 to 25 min. b) Schematic drawing of a single‐material 3D printed scaffolds made of either AHAT (blue) or HAT (orange). c,d) The swelling ratio of either AHAT or HAT 3D printed scaffolds with different crosslinking times, after 2 or 24 h of stimulus application. e) Young's modulus of the HAT scaffolds after 10 min of crosslinking and of the AHAT scaffolds after different crosslinking times (n = 3). f) Schematic drawing illustrating the post‐printing crosslinking treatment with 200 mm CaCl_2_ for 10 min of the biphasic 3D printed scaffold with 1 top layer of stable AHAT (blue) and 2 bottom layers of swellable HAT biomaterial ink (orange). g) The curvature of each scaffold crosslinked at different times, after 2 or 24 h of stimulus application. h) Representative images of the scaffolds crosslinked with different times, after 2 and 24 h of stimulus application. Scale bar for all pictures: 10 mm.

Since the curvature is affected by the elastic modulus of the biomaterial, the effect of varying the time of ionic crosslinking was investigated as another potential mechanism for controlling the degree of bending. First, the swelling ratio and compression modulus of the single‐material scaffolds made of either AHAT or HAT were studied after ionic crosslinking for different durations (**Figure** **6**a). For all crosslinking times (10, 15, 20, and 25 min), the AHAT scaffolds had a swelling ratio of 2.5 after 2 and 24 h (Figure 6c), while the HAT scaffolds crosslinked for 10 and 15 min showed differences in the swelling ratio from ≈3.5 at 2 h to ≈6 at 24 h (Figure 6d). After crosslinking for 20 and 25 min, the HAT scaffolds had a swelling ratio of ≈4 and ≈7 at 2 h, respectively, which was maintained after 24 h. Thus, the longer the crosslinking time, the faster the swelling of the HAT scaffolds. In addition, the compression tests revealed that the elastic modulus of AHAT (≈6–9 kPa) was not significantly affected by the crosslinking time. As expected from the results obtained earlier (Figure 3), regardless of the crosslinking time, the AHAT scaffolds had a significantly higher compression modulus (≈6–9 kPa) than the HAT group (≈2 kPa) (Figure 6e). With no significant differences either in the swelling ratio or stiffness of the AHAT part and a higher swelling ratio of the HAT as compared to AHAT in all conditions, the curvature of bilayered scaffolds crosslinked for different times is expected to be similar. The measured curvatures of the bilayered scaffolds (Figure 6f) after 24 h of swelling were, indeed, found to be independent of the crosslinking time (0.13–0.16 mm^−1^) (Figure 6g,h). For all four conditions, a significant increase in the curvature was observed from 2 to 24 h of swelling.

### 2.6 The Bilayer Approach Enables the Fabrication of Complex Self‐Bending 4D Printed Structures

**Figure 7:**
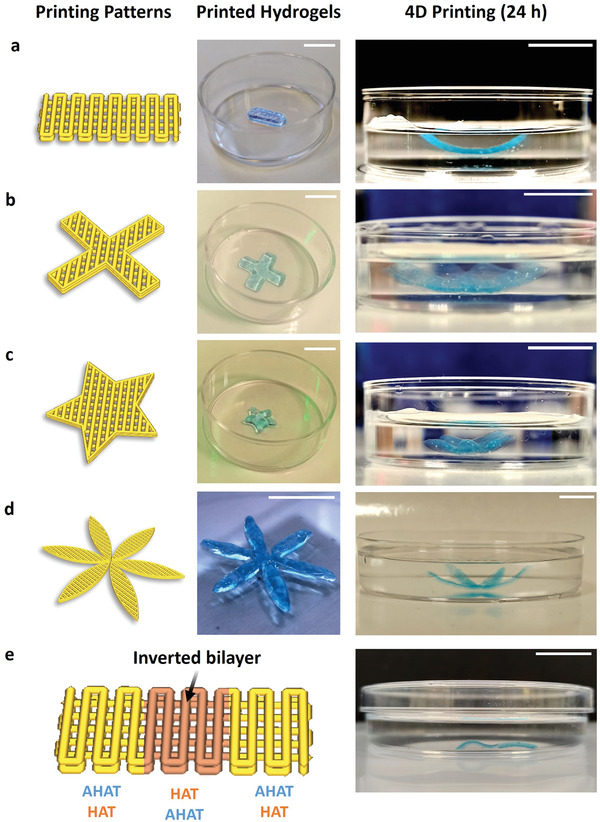
Structurally different constructs exhibiting different types of shape transformation. Representative images of the construct post‐printing, and after 24 h of stimulus application for the biphasic scaffolds made of one top layer AHAT and two bottom layers of HAT ink in the shape of: a) A (standard) rectangular construct, b) a cross‐shaped construct, c) a star‐shaped construct, d) a flower‐shaped construct, and e) a scaffold designed with alternating regions HAT/AHAT–AHAT/HAT– HAT/AHAT to create S‐shaped structures. The scale bars in all sub‐figures correspond to 10 mm.

To demonstrate the possibilities of this 4D printing approach, complex geometries other than rectangular scaffolds (**Figure** **7**a) were fabricated, including cross‐ (Figure 7b), star‐(Figure 7c), or flower‐shaped scaffolds (Figure 7d). The designed bilayers yielded out‐of‐plane structures upon swelling (see Video S1, Supporting Information). In all cases, the bilayers made of a bottom HAT layer and a top AHAT layer resulted in concave‐up bent structures. Inverting the order of the biomaterial inks in the alternating regions of the rectangular scaffold (HAT/AHAT–AHAT/HAT– HAT/AHAT; 1st layer/2nd layer) resulted in S‐shaped scaffolds (Figure 7e).

### 2.7 4D Bioprinting of Self‐Bending Bilayered Scaffolds Allows for the Localized Positioning of Cells in the Layers Mimicking Native Multicellular Curved Tissues

**Figure 8:**
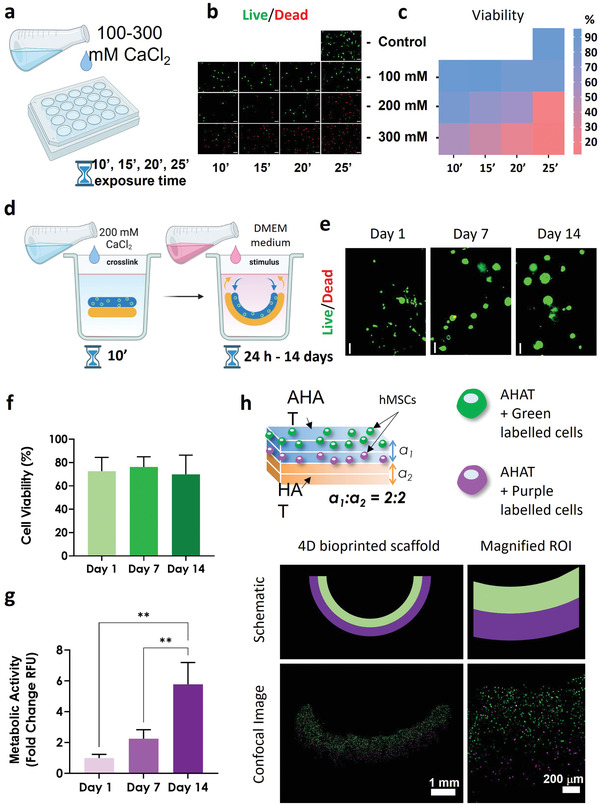
The effects of ionic crosslinking and 4D bioprinting on cell viability. a) Schematic drawing of the CaCl_2_ treatment on a monolayer of hMSCs. b) Live/dead fluorescent images showing alive cells in green and damaged/dead cells in red after exposure to different concentrations of CaCl_2_ at different times (emulating potential scaffold crosslinking treatments). c) The heat‐map plot quantifying the live/dead ratio in (b). d) Schematic drawing of the 4D bioprinting process from post‐printing ionic crosslinking with 200 mm CaCl_2_ for 10 min to 4D stimulus application via subsequent exposure to DMEM medium. e) The live/dead fluorescent representative images of the cell‐laden scaffolds (5 × 10^6^ cells mL^−1^) after 1, 7, and 14 days of stimulus application. f) The quantification of the % of viable cells from (e). g) The metabolic activity was determined by applying the Presto blue assay to the cell‐laden scaffolds at different time‐points. The data were normalized with respect to the day 1 values. h) Top: Schematic drawing of the biphasic scaffolds illustrating two cell‐laden AHAT layers and two cell‐free HAT layers. The cells from the top AHAT layer were labeled green, while the cells from the second AHAT layer were labeled red (colorized in purple for color‐blind aid); bottom: the images of the shape‐shifted scaffold 24 h post‐ionic crosslinking following bioprinting. The sub‐figure includes a schematic drawing of the colored‐labeled cell‐laden scaffolds and the corresponding confocal microscopy images at two different magnifications.

While crosslinking time did not have a significant effect on the curvature, the high ionic strength may adversely affect cell viability. We investigated the effects of exposure to different CaCl_2_ concentrations (100, 200, and 300 mm) for different times (10, 15, 20, and 25 min) on a monolayer of human mesenchymal stem/stromal cells (hMSCs) (**Figure** **8**a). Results indicated that cell viability is higher for lower calcium chloride concentrations and lower treatment times (Figure 8b,c). When using 100 mm CaCl_2_, the viability was affected after more than 20 min of exposure whereas for 200 mm CaCl_2_, the viability was affected after 15 min of exposure. Finally, with 300 mm CaCl_2_, the viability was affected for all the time‐points.

Next, bilayers were fabricated from the AHAT bioink (mixed with hMSCs) and the HAT ink (without cells). To ensure both alginate crosslinking as well as high cell viability, the bilayers were exposed to 200 mm CaCl_2_ for 10 min (Figure 8d). The constructs were then supplemented with DMEM‐based chondrogenic medium and cultured for 14 days. It was noted that different solutions (saline or DMEM) were found to have an effect on the swelling behavior of the inks and the curvature of the scaffolds (Figure S3, Supporting Information). The high viability was apparent up to 14 days after the application of the 4D printing stimulus (Figure 8e,f). Moreover, the metabolic activity of the cells, as measured with the Presto blue assay, was observed to increase from day 1 to day 14 (Figure 8g).

Then, we assessed whether the cells remain within the layer they were originally printed in after the shape transformation. The cells were pre‐labeled with either a green or red long‐term fluorescent marker before mixing them into the two different layers of the AHAT bioinks. The majority of the cells were found to be in their original layer 24 h post‐printing and after the development of the curvature (Figure 8h, Figure S5, Supporting Information, for better visualization).

### 2.8 Cartilage Tissue Engineering by Using the Self‐Curved 4D Bioprinted Constructs made with AHAT/HAT Bioinks: A Proof of Concept

**Figure 9:**
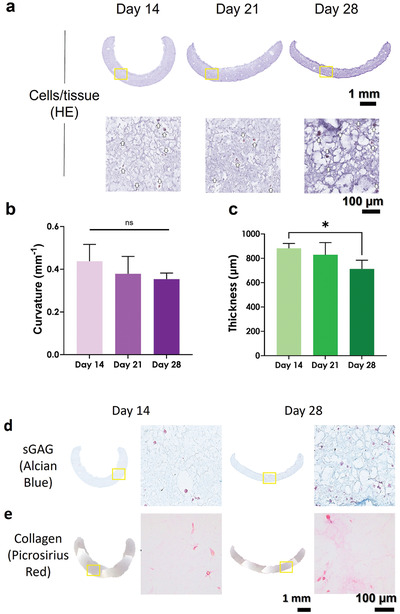
Self‐curved 4D bioprinted constructs made with AHAT/HAT bioinks for cartilage TE. Histology of 4D bioprinted constructs cultured under chondrogenic conditions. a) Representative histological images of hematoxylin‐eosin (HE staining) at two different magnifications at 14, 21, and 28 days of culture. The squared boxes represent a zoomed‐in region of interest. White arrows indicate the stained hMSCs. b) Curvature of the scaffold at 14, 21, and 28 days of culture. c) Thickness of the scaffolds at 14, 21, and 28 days of culture. Representative histological images of d) Alcian blue staining for sGAG in dark blue and e) Picrosirius red staining for collagen in dark pink at two different magnifications at 14 and 28 days of culture. The squared boxes represent a zoomed‐in region of interest.

4D bioprinted scaffolds containing hMSCs were cultured for 4 weeks in a chondrogenic medium to assess the scaffold's curvature and matrix development. After 28 days of culture, the cells were visible throughout the 4D bioprinted construct and its curvature was still present (**Figure** **9**a), albeit the curvature of the scaffolds exhibited a non‐significant time‐dependent decreasing trend (Figure 9b). Quantification of the thickness in the images of the HE staining of the scaffolds showed a significant thinning of the scaffolds during the 28 days of culture, perhaps due to the hydrogel being digested or dissolved over time (Figure 9c). Importantly, after 28 days in culture, the cells were observed to deposit cartilage‐containing molecules such as sulfated glycosaminoglycans (sGAGs) and collagen in the curved AHAT/HAT scaffolds, as visualized by Alcian blue and Picrosirius red staining (Figure 9d,e).

**Figure 10:**
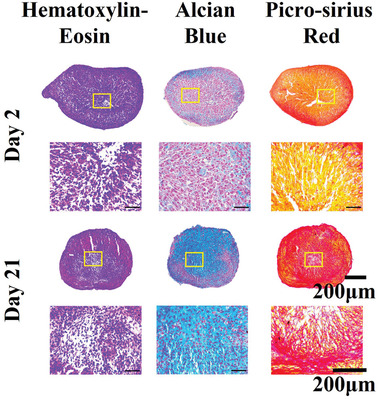
Chondrogenesis of human MSCs. Representative histological images of hematoxylin and eosin, alcian blue, and picrosirius‐red staining of MSC pellets cultured for 2 and 21 days in chondrogenic conditions at two different magnifications. The yellow box represents the magnified region of interest for the subfigures below each pellet.

MSCs cultured in microtissues or pellets (250 000 cells pellet^−1^) under the same chondrogenic conditions were used as positive controls. After 21 days of culture, the cells were observed to deposit cartilage‐like matrix such as sGAGs and collagen, visualized by HE, Alcian blue, and Picrosirius red staining (**Figure** **10**).

## 3 Discussion

In this study, we report an advanced 4D biofabrication method based on the differential swelling of a multi‐material hydrogel‐based bioink. This approach allowed for the controlled fabrication of bilayered scaffolds made from an alginate and hyaluronan‐tyramine composite ink (AHAT) and a hyaluronan‐tyramine alone ink (HAT), capable of self‐bending upon immersion in aqueous solution due to differential swelling. This study focuses on cartilage as the target tissue due to the curved nature of cartilage in locations such as the ear^[^ ^52^ ^]^ or the condyle.^[^ ^53^, ^54^ ^]^ In addition, cartilage, specifically articular cartilage, is a graded tissue with different layers that differ in cell phenotype and density, among other parameters.^[^ ^39^ ^]^ The incorporation of hMSCs in the composite hydrogel allowed for the fabrication of living, self‐bending scaffolds that could support multi‐layered high cell viability and cartilage‐like matrix deposition, demonstrating a proof‐of‐principle for the potential application of 4D bioprinting for cartilage TE.

Both inks used revealed their compatibility with extrusion‐based 3D printing. Namely, they exhibited a shear‐thinning behavior. HAT inks with similar H_2_O_2_ and HRP concentrations have been reported to show good printability during extrusion.^[^ ^50^ ^]^ The pre‐crosslinking of these inks with H_2_O_2_ was complete after ≈20 min, in line with the previous studies.^[^ ^55^ ^]^ The addition of alginate resulted in an AHAT composite ink with increased loss modulus, in line with previous studies where alginate was mixed with other biomaterial‐based inks, such as gelatin.^[^ ^56^, ^57^ ^]^ This might be due to the increased concentration of uncrosslinked polymer and a decreased ability to recover after exposure to high strain values, which has been associated with a lower structural integrity, but an increased extrusion uniformity.^[^ ^56^, ^57^ ^]^ Moreover, the addition of alginate also resulted in an increase in the damping factor. This, in turn, led to the need to increase the printing pressure (see experimental section/methods) to achieve similar printing patterns. Overall, the rheological characterization helped to predict the printability of both inks and to understand the differences in the values of the printing parameters (printing pressure) used for each ink. The inks used for the rheology measurements were cell‐free and, thus, the influence of cell encapsulation on the viscoelastic properties and the printability of the hydrogels were not investigated. Previous studies have reported that cell embedding can have an effect on the rheological properties of hydrogel‐based bioinks for cell densities varying between 2.5 × 10^6^ and 500 × 10^6^ cells mL^−1^.^[^ ^31^, ^58^, ^59^ ^]^ However, in these studies, the cells were added prior to preparing the ink, which can affect the pre‐crosslinking step of the ink and its final viscosity (and other rheological properties).^[^ ^31^ ^]^ The direct effects of the cells on the rheological properties of the bioink cannot, therefore, be de‐coupled from the effect of the cells on the chemical formulation of the ink itself. For this reason, we decided to mix the cell suspension with the AHAT ink after the enzymatic pre‐crosslinking was completed. In this study, no significant effects were observed on the printability between ink without cells and bioink containing cells. Nevertheless, further experiments to elucidate the direct effects that cells may have on the bioink rheological properties could provide valuable information for the bioprinting process.

Making use of the differential swelling properties of the two inks used, we studied the effects of a wide range of factors on scaffolds curvature, namely the infill density, printing angle, layer thickness, the CaCl_2_ crosslinking time, as well as the type of swelling solution. The HAT scaffolds swelled at least two times more than the AHAT composite. The lower liquid absorption by the AHAT composite hydrogel may be due to its higher crosslinking degree after gelling in presence of Ca^2+^ ions^[^ ^48^, ^49^, ^60^ ^]^ post‐printing. Previously, a decrease in the concentration of the crosslinking agent divinyl sulfone^[^ ^61^ ^]^ or a decrease in photocrosslinking time^[^ ^50^ ^]^ has been reported to cause a significant increase in the swelling ratios of HA hydrogels. Indeed, the alginate‐based ink was expected to have a swelling, and thus shape‐shifting behavior, that is dependent on Ca^2+^ cations up to saturation of the crosslinkable sites. Whereas a previous study reported that increasing the exposure time to 100 mm CaCl_2_ led to a decrease in the diameter of self‐folding tubes (or more pronounced folding) made from methacrylated alginate and HA,^[^ ^25^ ^]^ this behavior was not observed for the AHAT/HAT bilayer, where after immersion in 200 mm CaCl_2_ and increasing the crosslinking time of the constructs from 10 to 15, 20, and 25 min, no effect on the swelling behavior and mechanical properties were observed. A possible explanation for this difference could be the different CaCl_2_ concentrations used in each study. In addition, we noted that different solutions, such as saline or DMEM (alfa MEM was found to dissolve the scaffolds) had an effect on the swelling behavior of both inks and, consequently, on the curvature of the self‐bent scaffolds (Figure S3, Supporting Information). Although, both saline and DMEM used have a similar Ca^2+^ concentration (≈2 mm), the difference in swelling behavior may be due to the different ionic strengths of the swelling solutions. The ionic crosslinking of the AHAT composite and its higher total polymer concentration (2.5% for HAT vs 3.5% for AHAT) are also responsible for its higher compression modulus as compared to the HAT scaffolds. The swelling ratio and curvature experiments were carried out using a solution containing 0.9% NaCl with 2 mm CaCl_2_. It has, however, been reported that the storage modulus of sodium alginate hydrogels in this solution results in a significant decrease in both their compressive and shear moduli after 15 h, presumably due to the leaking of Ca^2+^ ions from the alginate.^[^ ^60^ ^]^ Therefore, the properties of our AHAT bioink during the swelling experiments as well as during the culture studies may deviate from those measured immediately after CaCl_2_ crosslinking and this is expected to influence shape‐shifting behavior. These results highlight the importance of the careful selection of the swelling, storing, and culture conditions of the scaffolds, particularly when the shape‐shifting is based on differential swelling properties.

Regarding the effects of infill density and printing angle on curvature, the findings revealed that infill densities lower than 40% produced fragile scaffolds, difficult to manipulate and characterize post‐printing. On the other hand, when the infill density was increased up to 60% the printed filaments started to touch without affecting the shape of the scaffold. A lower infill density (40%) resulted in a more pronounced bending. Several mechanistic explanations can be presented for this behavior. First, increasing the inter‐filamentous distance that is associated with a decreasing infill density causes the top and bottom surfaces of the scaffold to acquire a patterned profile, which could enhance the ability of the structure to bend, acting like a “backbone”. This is in line with previous research in which the surface patterning of a silk fibroin in paralleled regions has been reported to enhance the scaffold's self‐folding.^[^ ^18^ ^]^ Furthermore, an increased distance between adjacent filaments results in an increased surface area that is in direct contact with the swelling solvent. This mechanism could explain why scaffolds printed with the 40% infill density, which has a more “striated pattern”, showed a higher curvature from as early as 2 h after immersion.

Another design parameter to control curvature is the thickness of the different layers. A higher ratio of AHAT to HAT layers in the biphasic scaffolds resulted in a lower curvature. This could be due to physical limitations of the system in which the swelling force is not able to compensate for the ‘thick’ stiff AHAT to bend the structure further. Previous studies have pointed out that the selection of stimuli‐responsive materials is, in practice, a trade‐off between the amount of expansion and the material stiffness.^[^ ^62^ ^]^ Materials with a high elastic modulus often have small expansion values while the opposite holds true for materials with large expansion coefficients. On the other hand, a lower ratio of AHAT to HAT layers in the biphasic scaffold did not seem to significantly affect the obtained curvature. This behavior may be explained by the anisotropic swelling of the hydrogels, as the swelling behavior of different printed layers may not be identical. For instance, the first printed layer, located at the bottom of the structure, has a higher surface area in direct contact with the swelling solvent and, thus, may more easily expand towards that direction, whereas a layer located in the middle of the scaffold might exhibit a more pronounced swelling towards its sides. Interestingly, this effect may suppress the curvature obtained and even slow down the bending of the structures as it happens with the lower ratios of AHAT to HAT. In addition, increasing the layers can also have an effect on the curvature. This is in line with the previous research that has shown that thicker bilayer scaffolds made of polycaprolactone‐poly(glycerol sebacate) and methacrylated hyaluronic acid led to higher tube diameters (i.e., decreased curvature).^[^ ^63^ ^]^ These results underline the importance of the scaffold design for 4D printing, as relatively small changes in the 3D structure can have a significant impact on the shape‐change transformation.

Once printability, self‐bending, and curvature control were achieved, the 4D approach was further pushed to explore the fabrication of highly complex shapes, other than a rectangular bilayer, such as a cross, a star, or a flower. The successful curvature of these scaffolds clearly demonstrates the potential of 4D printing and it is important to highlight that the fabrication of these structures using standard 3D printing would be extremely challenging.

As a proof‐of‐concept, the incorporation of hMSCs into the 4D bioprinted scaffolds was then explored. Only the AHAT ink was mixed with cells, while the HAT phase of the scaffold was left cell‐free and was exclusively used to trigger the shape transformation. Leaving the HAT cell‐free allowed circumventing issues with the lower stability of these phase over time in culture, which may be related to the thinning of the scaffolds over time. Future studies could solve stability issues by adding a stabilizing secondary photocrosslinking step using the free tyramine groups.^[^ ^31^, ^50^, ^55^ ^]^ A high and sustained cell viability (≈75%) and an increase in the metabolic activity of the cells were measured in the 4D bioprinted constructs over 14 days of culture. The cell viability was further confirmed by histology after 28 days of culture. The applied printing pressure was compatible with the concept of the “biofabrication window” which describes the trade‐off between bioink printability and cell viability.^[^ ^64^, ^65^ ^]^ High cell viability has been reported in the extrusion bioprinting of hepatocarcinoma cell‐laden gelatin, using significantly higher pressures (1–2 bar) as well as for nozzles with smaller inner diameters (200 µm).^[^ ^58^ ^]^ Since viability can be affected by the extrusion, but also by exposure of cells to the encapsulation process, concentration of CaCl_2_, concentration of hydrogel, size of the constructs, as well as cell phenotype, the combination of all these parameters needs to be considered for each application.

Our approach also enables the spatial distribution of cells in the 4D bioprinted scaffolds, as the cells remained in their original layer post‐stimulus. This demonstrates the potential of 4D bioprinting to fabricate complex multilayer curved structures. For instance, this could be used to 4D bioprint a scaffold mimicking both the curvature and cell density typical of the native articular cartilage,^[^ ^39^ ^]^ or mimicking the organization of different cell types present in tissues such as blood vessels^[^ ^12^ ^]^ or the liver.^[^ ^66^ ^]^ Few studies^[^ ^14^ ^]^ have investigated 4D multicellular systems although none were actually stimulus‐triggered 4D bioprinting but rather tissue maturation‐dependent^[^ ^67^, ^68^ ^]^ or tissue self‐organization 3D bioprinting.^[^ ^69^ ^]^

In addition to controlling the spatial distribution of cells, we demonstrated that after 28 days of culture, the curvature of the scaffolds was still clearly present and cartilaginous matrix deposition was observed. Further research is needed to characterize the folding rate over time and the influence of scaffold biodegradation on the curvature (as it can be speculated that the faster degradation of the HAT material could unbend the scaffold), and to optimize tissue regeneration by generating a zonally organized structure. Previous studies have explored the addition of collagen,^[^ ^55^ ^]^ fibrin,^[^ ^70^ ^]^ fibroin,^[^ ^71^ ^]^ RGD peptides,^[^ ^72^ ^]^ or growth factors^[^ ^73^ ^]^ to alginate and hyaluronan‐based scaffolds to enhance the production of cell‐derived matrix, as well as application of different cell types or cell densities in different layers.^[^ ^39^, ^74^, ^75^, ^76^, ^77^ ^]^ These results pave the way for further experiments to study and optimize tissue development over time in 4D bioprinted self‐bending scaffolds.

## 4 Conclusions

In conclusion, a 4D bioprinting approach for the fabrication of bilayered scaffolds capable of self‐bending upon soaking in an aqueous solution was presented. In this study, a novel smart multi‐material system with biomaterials commonly used in TE was developed to enable the shape‐change transformation of 3D scaffolds into self‐bending constructs. The 4D bioprinted structures generated curved structures that were capable of maintaining a high cell viability and allowed for hMSC‐derived cartilaginous matrix deposition. Although still in its infancy, 4D bioprinting offers an unparalleled potential to unlock new strategies for cartilage TE and other structurally complex multicellular layered tissues.

## 5 Experimental Section

### Tyramine‐Functionalized Hyaluronan (HAT) Synthesis

HAT was synthesized as previously described.^[^ ^31^, ^50^, ^55^ ^]^ Briefly, HA (280–290 kDa, 5 mm carboxylic groups, Contipro Biotech S.R.O.) was functionalized via 4‐(4,6‐dimethoxy‐1,3,55‐triazin‐2‐yl)‐4‐mehtylmorpholini 2 µm chloride (DMTMM, TDI) amidation with tyramine by mixing at a stoichiometric ratio of 1:1:1. Functionalization was performed for 24 h at 37 °C. HAT was precipitated by adding ethanol (96% v/v) dropwise, isolated using Gooch filter no. 2, and dried into a powder. The degree of substitution was 6.6% as determined by absorbance reading at 275 nm (Multiskan GO Microplate Spectrophotometer; Thermo Fisher Scientific).

### Preparation of Biomaterial Inks Made from Either Tyramine‐Functionalized Hyaluronan (HAT) or Alginate Mixed with Tyramine‐Functionalized Hyaluronan (AHAT)

For the preparation of HAT ink, 2.5% w/v of HAT was reconstituted with 0.9% NaCl containing horseradish peroxidase enzyme (final [HRP] = 0.1 U mL^−1^) overnight at 4 °C in a shaker at 300 rpm (Thermal Shake lite, VWR, NL). The following day, the solution was mixed with H_2_O_2_ (final [H_2_O_2_] = 170 µm) via “female‐to‐female” luer‐lock adapter and kept in the dark for at least 30 min before using it for printing. For AHAT preparation, the same procedure was followed with the difference being the addition of 1% w/v of alginate (#4200001, Pronova UP LVG sodium alginate, Novamatrix, Sandvika, Norway) to the initial formulation.

### Rheological Characterization

The rheological properties of the AHAT and HAT inks were measured using two Anton Paar Physica MCR 501 rheometers (Anton Paar, Graz, Austria) equipped with a thermostatic hood and a Peltier element for temperature control. A cone‐plate geometry (diameter: 30 mm, angle: 1°) set at 21 °C was used to mimic the conditions of pre‐crosslinking and printing at room temperature. To prevent dehydration, a low viscosity mineral oil (Sigma‐Aldrich, viscosity ≈60 mPa s at 40 °C) was deposited on the perimeter of the samples and milli‐Q water was added in the circular groove surrounding the measuring platform to create a vapor‐saturated environment. Five different tests (i–v) were performed for each sample to measure the different rheological properties that can be essential in the different stages of 4D printing (Figure S4, Supporting Information): i) Each hydrogel was mixed with H_2_O_2_ and was immediately loaded on the rheometers. Pre‐crosslinking progress of the samples was recorded using a small‐amplitude oscillatory test, by measuring the time dependence of the storage and loss moduli (*G′* and *G″*, respectively). The duration of the test was set to 1 h (3600 s) with a 5 s interval between the consecutive measurements (720 measuring points). The frequency and strain were kept constant at 0.5 Hz (*π* rad s^−1^) and 0.5%, respectively. ii) The frequency dependence of the samples was investigated through a frequency sweep test by measuring *G′* and *G″* for 20 frequency values, logarithmically spaced in a range from 0.01 (0.02*π* rad s^−1^) to 10 Hz (20*π* rad s^−1^). Similar to the first test, the strain amplitude was kept constant at 0.5%. iii) Subsequently, a shear‐stress ramp from 0.01 Pa to 20 kPa with a slope of 20 points per decade was applied to the sample in order to measure its viscosity, *η*, under increasing shear. iv) Then a large amplitude oscillatory shear‐stress test from 1 Pa to 5 kPa (51 logarithmically spaced measuring points) was applied to the materials to identify their yield stress as the point where the *G′* and *G″* curves intersect. v) To allow for layer‐by‐layer 3D printing, the materials should be capable of quick recovering their solid‐like behavior after extrusion. To determine the strain‐dependent behavior of the inks with respect to their viscoelastic moduli, oscillatory thixotropy tests were performed. To mimic the printing process, the thixotropy/elastic recovery step test consisted of three steps: 1) an oscillatory 0.5% strain was applied to the material, 2) followed by a stress increase from 1 Pa to 5 kPa logarithmically in 51 steps (strain from 0.5% to 3.5 × 10^4^%), and 3) before returning to the original low oscillatory strain value. The frequency was set to 0.5 Hz for both the strain sweep and the oscillatory thixotropy tests. The first three measurements described above were performed on the same sample as consecutive tests of the same process, using the RheoPus software (Anton Paar, Graz, Austria). After the shear stress sweep, the samples had reached their breaking point and could not be used for further analyses. Therefore, for the last two measurements, a new, fully crosslinked sample of the same material was loaded on the rheometers. In total, three measurements were performed for each material, using newly prepared inks and employing both rheometers, yielding a total of six (*n* = 6) data sets per test. The values of the material parameters, *K* and *n* (Table 1) were determined for both inks by fitting the linear regime of the viscosity‐shear rate curve using the Ostwald‐de Waele power law equation^[^ ^51^ ^]^ for the apparent viscosity. The data were processed with MATLAB R2020b and were plotted using GraphPad Prism version 9.1.1. for Windows (GraphPad Software, SanDiego, California USA).

### Multi‐Material 4D Printing

HAT and AHAT biomaterial inks were used for 3D printing bilayered scaffolds with several designs: i) rectangular, ii) flower‐, iii) star‐, and iv) cross‐shaped. The CAD models of the scaffolds were designed using TinkerCAD (Autodesk, USA) and were exported as Standard Tessellation Language files (.stl). Then, the stl files were sliced using the Cura Ultimaker 4.7 and were exported as 3D objects (.3mf). The slicing parameters used were as follows: layer height = 0.25 mm; wall thickness = 0 mm for rectangular scaffolds and 0.25 mm for flower‐, star‐, and cross‐shaped scaffolds; infill density = 40, 50, or 60%; infill pattern = zig‐zag; infill line direction = [90°, 0°] and [0°, 90°] for rectangular scaffolds and [45°, 135°] for flower‐, star‐, and cross‐shaped scaffolds; speed = 10 mm s^−1^. The 3mf files were then imported to CAMotics v.1.2.0 and the g‐code of each design was generated. Finally, the g‐codes were imported to a BIO‐X 3D Bioprinter (Cellink, Sweden). A 25G conical nozzle (#7018391, Nordson Benelux, Netherlands) was used to print the bilayered scaffolds at room temperature. A pressure of 40 kPa was set for the HAT ink and 50 kPa for the AHAT ink. The printing speed was defined at 10 mm s^−1^ for both materials. Directly after printing, the scaffolds were crosslinked in 3 mL 200 mm CaCl_2_ (#C8106, Sigma, Netherlands) for 10 min. Then, the scaffolds were washed twice in 3 mL 0.9% NaCl for 5 min before allowing to swell in 0.9% NaCl containing 2 mm CaCl_2_ at room temperature for 24 h.

### Compression Test

After crosslinking, single‐material scaffolds with the dimensions of 10 × 4 × 1.5 mm^3^ were subjected to unconfined compression test performed at room temperature using a motorized compression test stand (ESM303, Mark‐10, USA) at a speed of 2 mm min^−1^. The stress was calculated as the load output divided by the measured contact surface of each scaffold and the strain was calculated based on the crosshead travel of the instrument. A preload value of 0.005 N was used to define the starting point of the compression tests. Finally, the compressive modulus, *E*, was determined by linear fitting (*R^2^* > 94%) in the region between 0–20% strain of the stress‐strain curve. The samples were prepared in triplicate (*n* = 3) for each condition.

### Swelling Analysis

For the determination of the mass swelling ratios (SR*_m_*) of the inks, single‐material test scaffolds were weighed using an analytical balance (AA‐160, Denver Instrument, US) directly after printing (*m*_print_). Subsequently, the scaffolds were crosslinked and washed before allowing to swell for up to 24 h to reach a swelling equilibrium. Each sample was weighed at two time points (i.e., 2 and 24 h) after printing to determine *m*_swollen_. The scaffolds were prepared in triplicate (*n* = 3) for each condition. The SR*_m_* was then determined by the following formula:

(1) $$SR_{m}=\frac{m_{swollen}-m_{print}}{m_{print}}$$

### Curvature Analysis

For the curvature analysis, the AHAT ink was dyed before printing with alcian blue (1:100 volume ratio dye:ink) to help visualize the scaffolds in saline solution. There was no need for dye when using the medium as swelling solution. Side‐view photos of the scaffolds in 35 mm petri‐dishes were acquired after 2 and 24 h of immersion in the swelling solvent. The images were processed using the Kappa plugin for Fiji.^[^ ^78^ ^]^ Three different curves were defined for each scaffold which were traced manually using 10 points per curve. The curvature (*κ*) was calculated using a B‐spline method where *ρ* is the radius of the circular arc that best approximates the curve of the scaffold. The average *κ* value of the three curves was selected as the curvature of the scaffold. Three scaffolds were used per condition (*n* = 3). The curvature, *κ*, was defined then as:

(2) $$\kappa =\frac{1}{\rho }$$

### Cell Expansion

Human bone marrow derived cells (MSCs) were purchased at passage 2 (Lonza Bioscience, Netherlands) and were expanded to passage 4 before further use in the expansion medium:^[^ ^79^ ^]^ *α*MEM (#10712124, Thermo Fisher, Netherlands), 1.5 µg mL^−1^ fungizone (#11510496, Thermo Fisher, Netherlands), 50 µg mL^−1^ gentamicin (#11520506, Thermo Fisher, Netherlands), 10% v/v FBS (#15517, Thermo Fisher, Netherlands), 100 µm ascorbic acid (#A8960, Sigma, Netherlands), and 1 ng mL^−1^ fibroblast growth factor‐2 (#PHP105, Bio‐Rad, Netherlands). The medium was refreshed twice weekly. The cells at passage 5 were used for bioprinting.

### Effect of CaCl_2_ on hMSCs

Before the start of the bioprinting experiments, the effects of CaCl2 on hMSCs were investigated. Solutions of 100, 200, and 300 mm of CaCl_2_ in saline were prepared and sterile‐filtered (0.2 µm syringe filters). hMSCs cultured in monolayers in a 48‐well plate for 14 days were washed twice with 1 × PBS for 5 min. Subsequently, hMSCs were incubated at room temperature with 100 µL of the aforementioned CaCl_2_ solutions for 10, 15, 20, and 25 min (*n* = 3 per time‐point and concentration value). The wells in saline without CaCl_2_ were used as the controls (*n* = 3).

### 4D Bioprinting

The bioprinter was wiped with 70% ethanol and UV‐sterilized before moving it into the biosafety cabinet. The HAT and alginate powders were UV‐sterilized and the solutions were sterile‐filtered prior to biomaterial ink formulation. Once the inks were ready to use, the AHAT ink was gently mixed with a suspension of ≈5 × 10^6^ cells mL^−1^ MSCs in a volume of medium equal to 10% of the total bioink volume, while the HAT ink was used acellular. Bilayer scaffolds were bioprinted as described before in 12‐well plates. Subsequently, 3 mL of sterile 200 mm CaCl_2_ was added to the scaffold‐containing wells for 10 min. The solution was then discarded and the scaffolds were washed twice with 3 mL of 0.9% NaCl for 5 min. Finally, 3 mL of the expansion medium, made with DMEM (#11594446, Thermo fisher) due to instability of the HAT material in *α*MEM (data not shown), were added to each well. The samples were placed in an incubator at 37 °C, 5% CO2, and 90% humidity. The medium was refreshed twice weekly.

### Chondrogenic Conditions

To explore the chondrogenic differentiation of hMSC in 4D bioprinted scaffolds, the constructs were cultured in chondrogenic medium:^[^ ^79^ ^]^ DMEM (#11574456, Thermo Fisher, Netherlands), 1x ITS+ (#11593560, Thermo Fisher, Netherlands), 40 µg mL^−1^ L‐Proline (#P5607, Sigma, Netherlands), 1 mm sodium pyruvate (#11530396, Thermo Fisher, Netherlands), 1.5 µg mL^−1^ fungizone (#11510496, Thermo Fisher, Netherlands), 50 µg mL^−1^ gentamicin (#11520506, Thermo Fisher, Netherlands), and freshly added to make, 10 ng mL^−1^ Transforming growth factor‐*β*1 (#10364313, Thermo Fisher, Netherlands), 100 nm dexamethasone (#D4902, Sigma, Netherlands), and 100 µm ascorbic acid (#A8960, Sigma, Netherlands). DMEM contains 2 mm CaCl_2_ among other salts. Six pellets of 250 000 MSCs cultured in the chondrogenic medium were used as the controls for positive chondrogenesis.

### Live/Dead Assay

The cell viability of the bioprinted constructs was assessed at days 1, 7, and 14 after culture using live/dead staining (#12353643, LIVE/DEAD Viability/Cytotoxicity Kit, ThermoFisher, Delft, The Netherlands). Briefly, the samples were washed twice with saline for 5 min before supplementing the scaffolds with 2 mm ethidium homodimer‐1 (red, for dead cells) and 5 mm calcein‐AM (green, for live cells) in saline. The samples were allowed to incubate for 30 min at room temperature in the dark before being washed twice in saline and being imaged under a fluorescent microscope (ZOE fluorescent cell imager, Biorad, Delft, The Netherlands). For 2D monolayers of hMSCs exposed to CaCl_2_, PBS was used instead of saline.

### Metabolic Activity

The PrestoBlue assays were used after 1, 7, and 14 days of culture to measure the metabolic activity of the cells. The assay was performed according to manufacturer's instructions (#12083745, Thermo Fisher, Netherlands). Briefly, PrestoBlue was added to 1 mL fresh medium at a final concentration of 10%. The scaffolds (*n* = 3) were incubated in the solution for 1 h at 37 °C (5% CO_2_, 90% humidity). Subsequently, 100 µL of the medium for each sample was transferred into a 96‐well plate in triplicates (*n* = 3). The fluorescence (Relative Fluorescent Unit, RFU) was read at 570 nm using a microplate reader (PerkinElmer, Massachusetts, US). The corrected RFU was calculated by subtracting the average blank well value (PrestoBlue with medium) from the measured values of the scaffold‐containing wells and were presented as mean ± standard deviation.

### Histological Analysis

Samples were taken after 2, 3, and 4 weeks of culture (*n* = 3), which were subsequently fixed overnight at 4 °C with a tissue–fixing solution volume ratio of 1:20 in a solution containing 2% PFA (#252549, Sigma, Netherlands), 2.5% Glutaraldehyde (#8206030100, Sigma, Netherlands), 0.5% Cetylpyridinum chloride (#8400080100, Sigma, Netherlands), and 50 mm CaCl_2_ in 0.9% NaCl solution. Then, the scaffolds were washed twice with saline and were dehydrated with serial dilutions of ethanol and xylene prior to paraffin wax‐embedding (#39601006, Paraplast, Leica, Netherlands). The samples were sectioned at a thickness of 6 µm. The sections were stained with hematoxylin (HHS32, Sigma, The Netherlands) and eosin (HT110232, Sigma, The Netherlands) to examine their cell distribution, with 1% Alcian Blue at pH 1 (TMS‐010‐C, Sigma, The Netherlands) to analyze their sGAG content, and with Picrosirius Red (365548, Sigma, The Netherlands) for collagen deposition. The stained histological slices were imaged under a DM500 optical Leica microscope.

### Statistical Analysis

The samples were assessed in triplicate for each condition and the acquired data were presented as mean value ± standard deviation. Two‐way ANOVA followed by multiple comparisons test was performed for grouped data, using GraphPad Prism version 9.1.1. for Windows (GraphPad Software, San Diego, California USA). When only two datasets were compared, the Welch's t‐test was used. A *p* value < 0.05 was considered significant. ∗ *p* < 0.05, ∗∗ *p* < 0.01, ∗∗∗ *p* < 0.001, ∗∗∗∗ *p* < 0.0001.

## Conflict of Interest

The authors declare no conflict of interest.

## Supporting information

Supporting Information

Supporting Video 1

## Data Availability

The data that support the findings of this study are available from the corresponding author upon reasonable request.

## References

[1] Y. Chen , J. Zhang , X. Liu , S. Wang , J. Tao , Y. Huang , W. Wu , Y. Li , K. Zhou , X. Wei , S. Chen , X. Li , X. Xu , L. Cardon , Z. Qian , M. Gou , Sci. Adv. 2020, 6, eaba7406. 32537512 10.1126/sciadv.aba7406 PMC7269646

[2] M. Costantini , S. Testa , P. Mozetic , A. Barbetta , C. Fuoco , E. Fornetti , F. Tamiro , S. Bernardini , J. Jaroszewicz , W. Święszkowski , M. Trombetta , L. Castagnoli , D. Seliktar , P. Garstecki , G. Cesareni , S. Cannata , A. Rainer , C. Gargioli , Biomaterials 2017, 131, 98. 28388499 10.1016/j.biomaterials.2017.03.026

[3] A. Lee , A. R. Hudson , D. J. Shiwarski , J. W. Tashman , T. J. Hinton , S. Yerneni , J. M. Bliley , P. G. Campbell , A. W. Feinberg , Science 2019, 365, 482. 31371612 10.1126/science.aav9051

[4] J. Groll , T. Boland , T. Blunk , J. A. Burdick , D.‐W. Cho , P. D. Dalton , B. Derby , G. Forgacs , Q. Li , V. A. Mironov , L. Moroni , M. Nakamura , W. Shu , S. Takeuchi , G. Vozzi , T. B. F. Woodfield , T. Xu , J. J. Yoo , J. Malda , Biofabrication 2016, 8, 013001. 26744832 10.1088/1758-5090/8/1/013001

[5] T. Jiang , J. G. Munguia‐Lopez , S. Flores‐Torres , J. Kort‐Mascort , J. M. Kinsella , Appl. Phys. Rev. 2019, 6, 011310.

[6] X. Li , B. Liu , B. Pei , J. Chen , D. Zhou , J. Peng , X. Zhang , W. Jia , T. Xu , Chem. Rev. 2020, 120, 10793. 32902959 10.1021/acs.chemrev.0c00008

[7] W. L. Ng , J. M. Lee , M. Zhou , Y.‐W. Chen , K.‐X. A. Lee , W. Y. Yeong , Y.‐F. Shen , Biofabrication 2020, 12, 022001. 31822648 10.1088/1758-5090/ab6034

[8] W. Sun , B. Starly , A. C. Daly , J. A. Burdick , J. Groll , G. Skeldon , W. Shu , Y. Sakai , M. Shinohara , M. Nishikawa , J. Jang , D.‐W. Cho , M. Nie , S. Takeuchi , S. Ostrovidov , A. Khademhosseini , R. D. Kamm , V. Mironov , L. Moroni , I. T. Ozbolat , Biofabrication 2020, 12, 022002. 32031083 10.1088/1758-5090/ab5158

[9] A. Schwab , R. Levato , M. D'Este , S. Piluso , D. Eglin , J. Malda , Chem. Rev. 2020, 120, 11028. 32856892 10.1021/acs.chemrev.0c00084 PMC7564085

[10] H.‐J. Jeong , H. Nam , J. Jang , S.‐J. Lee , Bioengineering 2020, 7, 32. 32244491 10.3390/bioengineering7020032 PMC7357036

[11] A. A. Giannopoulos , D. Mitsouras , S.‐J. Yoo , P. P. Liu , Y. S. Chatzizisis , F. J. Rybicki , Nat. Rev. Cardiol. 2016, 13, 701. 27786234 10.1038/nrcardio.2016.170

[12] D. Richards , J. Jia , M. Yost , R. Markwald , Y. Mei , Ann. Biomed. Eng. 2017, 45, 132. 27230253 10.1007/s10439-016-1653-z PMC5124424

[13] T. Van Manen , S. Janbaz , K. M. B. Jansen , A. A. Zadpoor , Commun. Mater. 2021, 2, 56.

[14] B. Gao , Q. Yang , X. Zhao , G. Jin , Y. Ma , F. Xu , Trends Biotechnol. 2016, 34, 746. 27056447 10.1016/j.tibtech.2016.03.004

[15] Y.‐C. Li , Y. S. Zhang , A. Akpek , S. R. Shin , A. Khademhosseini , Biofabrication 2016, 9, 012001. 27910820 10.1088/1758-5090/9/1/012001

[16] T. Van Manen , S. Janbaz , A. A. Zadpoor , Mater. Horiz. 2017, 4, 1064. 29308207 10.1039/c7mh00269f PMC5735361

[17] W. J. Hendrikson , J. Rouwkema , F. Clementi , C. A. Van Blitterswijk , S. Farè , L. Moroni , Biofabrication 2017, 9, 031001. 28726680 10.1088/1758-5090/aa8114

[18] S. H. Kim , Y. B. Seo , Y. K. Yeon , Y. J. Lee , H. S. Park , Md. T. Sultan , J. M. Lee , J. S. Lee , O J. Lee , H. Hong , H. Lee , O. Ajiteru , Y. J. Suh , S.‐H. Song , K.‐H. Lee , C. H. Park , Biomaterials 2020, 260, 120281. 32858503 10.1016/j.biomaterials.2020.120281

[19] G. H. Yang , W. Kim , J. Kim , G. Kim , Theranostics 2021, 11, 48. 33391460 10.7150/thno.50794 PMC7681100

[20] V. Du , N. Luciani , S. Richard , G. T. Mary , C. Gay , F. §. O. Mazuel , M. Reffay , P. Menasché , O. Agbulut , C. Wilhelm , Nat. Commun. 2017, 8, 400. 28900152 10.1038/s41467-017-00543-2 PMC5596024

[21] Y. Luo , X. Lin , B. Chen , X. Wei , Biofabrication 2019, 11, 045019. 31394520 10.1088/1758-5090/ab39c5

[22] Y. bin Lee , O. Jeon , S. J. Lee , A. Ding , D. Wells , E. Alsberg , Adv. Funct. Mater. 2021, 31, 2010104. 34335134 10.1002/adfm.202010104 PMC8323845

[23] A. Ding , S. J. Lee , R. Tang , K. L. Gasvoda , F. He , E. Alsberg , Small 2022, 18, 2202196. 10.1002/smll.202202196 PMC9463124 35973946

[24] A. Ding , O. Jeon , D. Cleveland , K. L. Gasvoda , D. Wells , S. J. Lee , E. Alsberg , Adv. Mater. 2022, 34, 2109394. 10.1002/adma.202109394 PMC9012690 35065000

[25] A. Kirillova , R. Maxson , G. Stoychev , C. T. Gomillion , L. Ionov , Adv. Mater. 2017, 29, 1703443. 10.1002/adma.201703443 29024044

[26] C. Vergallo , L. Dini , Sustainability 2018, 10, 2776.

[27] M. G. Li , X. Y. Tian , X. Chen , Artif. Organs 2011, 35, 741. 21752034 10.1111/j.1525-1594.2010.01193.x

[28] M. Klak , M. Gomółka , T. Dobrzański , G. Tymicki , P. Cywoniuk , P. Kowalska , K. Kosowska , T. Bryniarski , A. Berman , A. Dobrzyå„ , J. Idaszek , PLoS One 2020, 15, e0235052. 32584858 10.1371/journal.pone.0235052 PMC7316267

[29] C. Huang , Z. Wang , D. Quinn , S. Suresh , K. J. Hsia , Proc. Natl. Acad. Sci. USA 2018, 115, 12359. 30455311 10.1073/pnas.1811296115 PMC6298086

[30] R. Kempaiah , Z. Nie , J. Mater. Chem. B 2014, 2, 2357. 32261408 10.1039/c3tb21462a

[31] D. Petta , A. R. Armiento , D. Grijpma , M. Alini , D. Eglin , M. D'Este , Biofabrication 2018, 10, 044104. 30188324 10.1088/1758-5090/aadf58

[32] M. N. Collins , C. Birkinshaw , Carbohydr. Polym. 2013, 92, 1262. 23399155 10.1016/j.carbpol.2012.10.028

[33] S. Stichler , T. Böck , N. Paxton , S. Bertlein , R. Levato , V. Schill , W. Smolan , J. Malda , J. Teßmar , T. Blunk , J. Groll , Biofabrication 2017, 9, 044108. 28906257 10.1088/1758-5090/aa8cb7

[34] A. Abbadessa , V. H. M. Mouser , M. M. Blokzijl , D. Gawlitta , W. J. A. Dhert , W. E. Hennink , J. Malda , T. Vermonden , Biomacromolecules 2016, 17, 2137. 27171342 10.1021/acs.biomac.6b00366 PMC4931898

[35] K.‐C. Hung , C.‐S. Tseng , L.‐G. Dai , S.‐H. Hsu , Biomaterials 2016, 83, 156. 26774563 10.1016/j.biomaterials.2016.01.019

[36] J. Kundu , J.‐H. Shim , J. Jang , S.‐W. Kim , D.‐W. Cho , J. Tissue Eng. Regener. Med. 2015, 9, 1286. 10.1002/term.1682 23349081

[37] K. Markstedt , A. Mantas , I. Tournier , H. Martínez Ávila , D. Hägg , P. Gatenholm , Biomacromolecules 2015, 16, 1489. 25806996 10.1021/acs.biomac.5b00188

[38] M. Müller , E. Öztürk , Ø. Arlov , P. Gatenholm , M. Zenobi‐Wong , Ann. Biomed. Eng. 2017, 45, 210. 27503606 10.1007/s10439-016-1704-5

[39] A. Dimaraki , P. J. Díaz‐Payno , M. Minneboo , M. Nouri‐Goushki , M. Hosseini , N. Kops , R. Narcisi , M. J. Mirzaali , G. J. V. M. Van Osch , L. E. Fratila‐Apachitei , A. A. Zadpoor , Appl. Sci. 2021, 11, 7821.

[40] P. Rastogi , B. Kandasubramanian , Biofabrication 2019, 11, 042001. 31315105 10.1088/1758-5090/ab331e

[41] E. Axpe , M. Oyen , Int. J. Mol. Sci. 2016, 17, 1976. 27898010 10.3390/ijms17121976 PMC5187776

[42] B. Wang , P. J. Díaz‐Payno , D. C. Browe , F. E. Freeman , J. Nulty , R. Burdis , D. J. Kelly , Acta Biomater. 2021, 128, 130. 33866035 10.1016/j.actbio.2021.04.016

[43] W. J. C. M. Marijnissen , G. J. V. M. Van Osch , J. Aigner , S. W. van der Veen , A. P. Hollander , H. T. L. Verwoerd‐Verhoef , J. A. N. Verhaar , Biomaterials 2002, 23, 1511. 11833491 10.1016/s0142-9612(01)00281-2

[44] H. J. Hauselmann , K. Masuda , E. B. Hunziker , M. Neidhart , S. S. Mok , B. A. Michel , E. J. Thonar , Am. J. Physiol.: Cell Physiol. 1996, 271, C742. 10.1152/ajpcell.1996.271.3.C742 8843703

[45] N. Hendrijantini , Dent. J. 2019, 52, 36.

[46] D. M. W. Anderson , W. G. Brydon , M. A. Eastwood , D. M. Sedgwick , Food Addit. Contam. 1991, 8, 237. 1778263 10.1080/02652039109373974

[47] A. R. Kim , J. H. Hwang , H. M. Kim , H. N. Kim , J. E. Song , Y. I. Yang , K. H. Yoon , D. Lee , G. Khang , J. Biomater. Sci., Polym. Ed. 2013, 24, 1084. 23683040 10.1080/09205063.2012.735100

[48] S. N. Pawar , K. J. Edgar , Biomaterials 2012, 33, 3279. 22281421 10.1016/j.biomaterials.2012.01.007

[49] N. E. Simpson , C. L. Stabler , C. P. Simpson , A. Sambanis , I. Constantinidis , Biomaterials 2004, 25, 2603. 14751746 10.1016/j.biomaterials.2003.09.046

[50] D. Petta , D. W. Grijpma , M. Alini , D. Eglin , M. D'Este , ACS Biomater. Sci. Eng. 2018, 4, 3088. 33435028 10.1021/acsbiomaterials.8b00416

[51] W. Ostwald , Kolloid‐Z. 1929, 47, 176.

[52] A. J. Lin , J. L. Bernstein , J. A. Spector , Curr. Surg. Rep. 2018, 6, 4.

[53] G. M. Cunniffe , P. J. Díaz‐Payno , E. J. Sheehy , S. E. Critchley , H. V. Almeida , P. Pitacco , S. F. Carroll , O. R. Mahon , A. Dunne , T. J. Levingstone , C. J. Moran , R. T. Brady , F. J. O'brien , P. A. J. Brama , D. J. Kelly , Biomaterials 2019, 188, 63. 30321864 10.1016/j.biomaterials.2018.09.044

[54] D. C. Browe , P. J. Díaz‐Payno , F. E. Freeman , R. Schipani , R. Burdis , D. P. Ahern , J. M. Nulty , S. Guler , L. D. Randall , C. T. Buckley , P. A. J. Brama , D. J. Kelly , Acta Biomater. 2022, 143, 266. 35278686 10.1016/j.actbio.2022.03.009

[55] A. Schwab , C. Hélary , R. G. Richards , M. Alini , D. Eglin , M. D'este , Mater. Today Bio 2020, 7, 100058. 10.1016/j.mtbio.2020.100058 PMC7317236 32613184

[56] T. Gao , G. J. Gillispie , J. S. Copus , A. K. Pr , Y.‐J. Seol , A. Atala , J. J. Yoo , S. J. Lee , Biofabrication 2018, 10, 034106. 29923501 10.1088/1758-5090/aacdc7 PMC6040670

[57] I. Gorroñogoitia , U. Urtaza , A. Zubiarrain‐Laserna , A. Alonso‐Varona , A. M. Zaldua , Polymers 2022, 14, 354. 35054760 10.3390/polym14020354 PMC8778016

[58] T. Billiet , E. Gevaert , T. De Schryver , M. Cornelissen , P. Dubruel , Biomaterials 2014, 35, 49. 24112804 10.1016/j.biomaterials.2013.09.078

[59] A. Skardal , J. Zhang , G. D. Prestwich , Biomaterials 2010, 31, 6173. 20546891 10.1016/j.biomaterials.2010.04.045

[60] M. A. Leroux , F. Guilak , L. A. Setton , J. Biomed. Mater. Res. 1999, 47, 46. 10400879 10.1002/(sici)1097-4636(199910)47:1<46::aid-jbm6>3.0.co;2-n

[61] C. B. Shah , S. M. Barnett , J. Appl. Polym. Sci. 1992, 45, 293.

[62] T. van Manen , A. A. Zadpoor , Theoretical stiffness limits of 4D printed self‐folding metamaterials, arXiv, 2021, 10.48550/ARXIV.2106.05790.

[63] I. Apsite , G. Constante , M. Dulle , L. Vogt , A. Caspari , A. R. Boccaccini , A. Synytska , S. Salehi , L. Ionov , Biofabrication 2020, 12, 035027. 32434153 10.1088/1758-5090/ab94cf

[64] N. Paxton , W. Smolan , T. Böck , F. Melchels , J. Groll , T. Jungst , Biofabrication 2017, 9, 044107. 28930091 10.1088/1758-5090/aa8dd8

[65] K. A. Deo , K. A. Singh , C. W. Peak , D. L. Alge , A. K. Gaharwar , Tissue Eng., Part A 2020, 26, 318. 32079490 10.1089/ten.tea.2019.0298 PMC7480731

[66] R. Taymour , D. Kilian , T. Ahlfeld , M. Gelinsky , A. Lode , Sci. Rep. 2021, 11, 5130. 33664366 10.1038/s41598-021-84384-6 PMC7933206

[67] C. Norotte , F. S. Marga , L. E. Niklason , G. Forgacs , Biomaterials 2009, 30, 5910. 19664819 10.1016/j.biomaterials.2009.06.034 PMC2748110

[68] F. Xu , J. Celli , I. Rizvi , S. Moon , T. Hasan , U. Demirci , Biotechnol. J. 2011, 6, 204. 21298805 10.1002/biot.201000340 PMC3780785

[69] J. A. Brassard , M. Nikolaev , T. Hasan , M. Hofer , M. P. Lutolf , Nat. Mater. 2021, 20, 22. 32958879 10.1038/s41563-020-00803-5

[70] S.‐H. Park , S. R. Park , S. I. Chung , K. S. Pai , B.‐H. Min , Artif. Organs 2005, 29, 838. 16185347 10.1111/j.1525-1594.2005.00137.x

[71] R. Ziadlou , S. Rotman , A. Teuschl , E. Salzer , A. Barbero , I. Martin , M. Alini , D. Eglin , S. Grad , Mater. Sci. Eng., C 2021, 120, 111701. 10.1016/j.msec.2020.111701 33545860

[72] F. Z. Cui , W. M. Tian , S. P. Hou , Q. Y. Xu , I. ‐ S. Lee , J. Mater. Sci.: Mater. Med. 2006, 17, 1393. 17143772 10.1007/s10856-006-0615-7

[73] R. Mhanna , J. Becher , M. Schnabelrauch , R. L. Reis , I. Pashkuleva , Adv. Biosyst. 2017, 1, 1700043. 10.1002/adbi.201700043 32646173

[74] S. Talukdar , Q. T. Nguyen , A. C. Chen , R. L. Sah , S. C. Kundu , Biomaterials 2011, 32, 8927. 21906805 10.1016/j.biomaterials.2011.08.027 PMC3183393

[75] R. L. Mauck , C. C. ‐ B. Wang , E. S. Oswald , G. A. Ateshian , C. T. Hung , Osteoarthritis Cartilage 2003, 11, 879. 14629964 10.1016/j.joca.2003.08.006

[76] G. M. Williams , T. J. Klein , R. L. Sah , Acta Biomater. 2005, 1, 625. 16701843 10.1016/j.actbio.2005.07.009

[77] A. Troken , N. Marion , S. Hollister , J. Mao , Proc. Inst. Mech. Eng., Part H 2007, 221, 429. 10.1243/09544119JEIM288 17822145

[78] J. Schindelin , I. Arganda‐Carreras , E. Frise , V. Kaynig , M. Longair , T. Pietzsch , S. Preibisch , C. Rueden , S. Saalfeld , B. Schmid , J.‐Y. Tinevez , D. J. White , V. Hartenstein , K. Eliceiri , P. Tomancak , A. Cardona , Nat. Methods 2012, 9, 676. 22743772 10.1038/nmeth.2019 PMC3855844

[79] R. Narcisi , W. J. L. M. Koevoet , G. J. V. M. van Osch , in Osteoporosis and Osteoarthritis. Methods in Molecular Biology (Eds: A. J. van Wijnen , M. S. Ganshina ), vol. 2221, Humana, New York, NY, 2021, pp. 15–28. 10.1007/978-1-0716-0989-7_2 32979195

