# Supplementary material for: Swelling‐Dependent Shape‐Based Transformation of a Human Mesenchymal Stromal Cells‐Laden 4D Bioprinted Construct for Cartilage Tissue Engineering
Source: Adv Healthc Mater. 2022 Nov 17;12(2):2201891. doi: 10.1002/adhm.202201891 (PMC11468569; doi:10.1002/adhm.202201891)
Supplement: Supplementary file 1 — Supporting Information [file ADHM-12-2201891-s001.pdf]

ADVANCED HEALTHCARE MATERIALS

Supporting Information

for *Adv. Healthcare Mater.*, DOI 10.1002/adhm.202201891

Swelling-Dependent Shape-Based Transformation of a Human Mesenchymal Stromal Cells-Laden 4D Bioprinted Construct for Cartilage Tissue Engineering

Pedro J. Díaz-Payno, Maria Kalogeropoulou, Iain Muntz, Esther Kingma, Nicole Kops, Matteo D'Este, Gijsje H. Koenderink, Lidy E. Fratila-Apachitei, Gerjo J. V. M. van Osch* and Amir A. Zadpoor**

Supporting Information

Swelling-dependent shape-based transformation of a hMSC-laden 4D bioprinted construct for cartilage tissue engineering

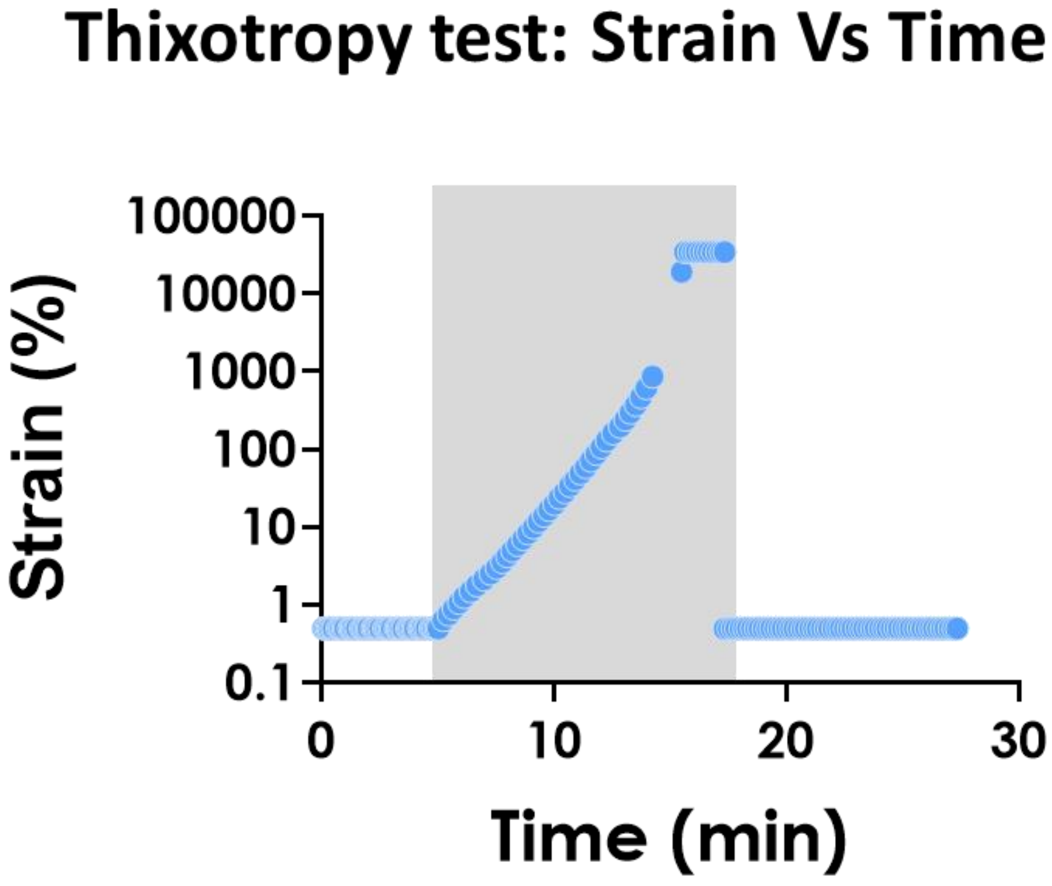

Figure S1. Thixotropy test control. Representative graft showing the Strain Vs time values for the thixotropy test from Figure 2g,h. The first and third step (non-shadowed) was kept at a constant 0.5 % strain, while in the second step (shadowed area) the stress was increased from 1 Pa to 5 kPa logarithmically in 51 steps.

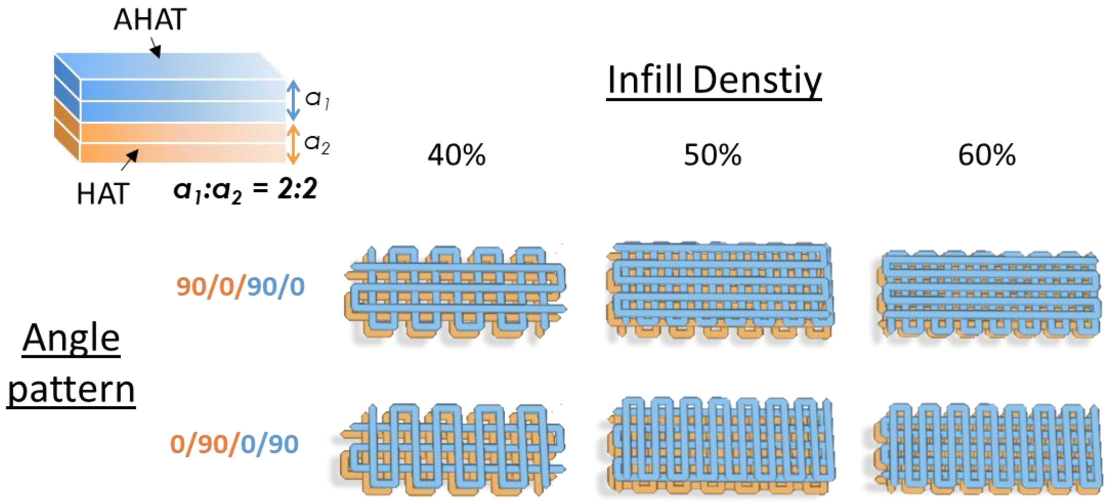

Figure S2. Schematic of 3D printing designs. Design of the AHAT: HAT layers 2:2 with different infill densities: 40, 50, and 60%; and the different angle pattern of each layer: 90/0/90/0° and 0/90/0/90°.

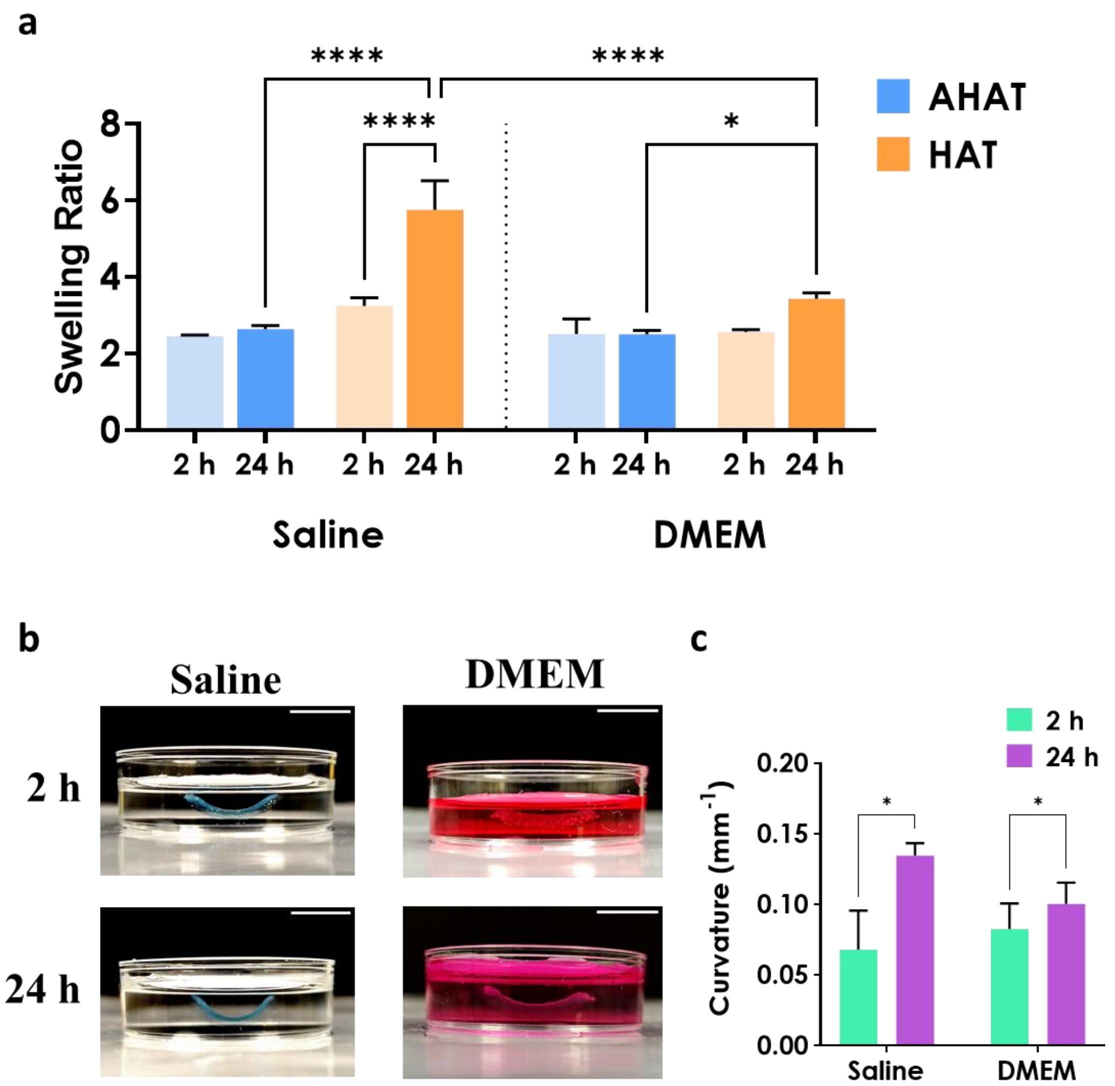

Figure S3. Swelling and shape-change depends on swelling solution. a) The swelling ratios of the AHAT (blue) and HAT (orange) printed scaffolds after 2 and 24 h of immersion in either saline (left) or DMEM (right). b) Representative images of the bilayer scaffolds made of AHAT:HAT 2:1 after 2 and 24 h of swelling in either saline (left) or DMEM (right). c) The degree of curvature measured from b) shows an increasing degree of curvature in both swelling solutions.

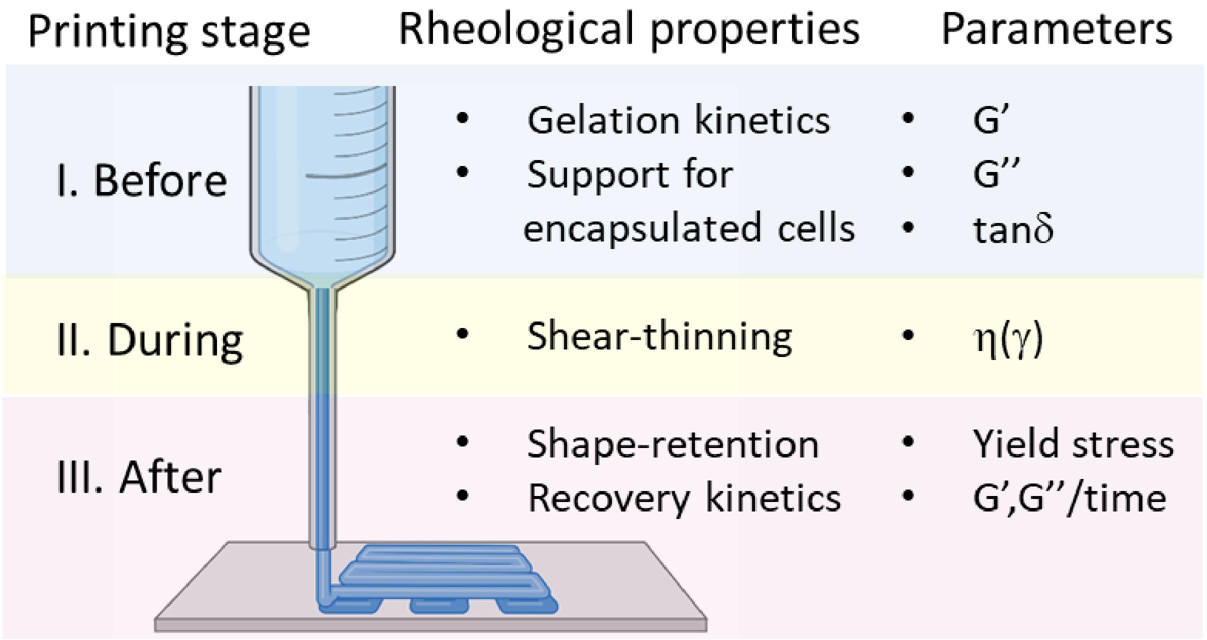

Figure S4. 3D printing and rheology. The different stages of the 3D/4D printing process: before printing, during printing, and after printing; and the relevant rheological properties and main parameters of each stage.

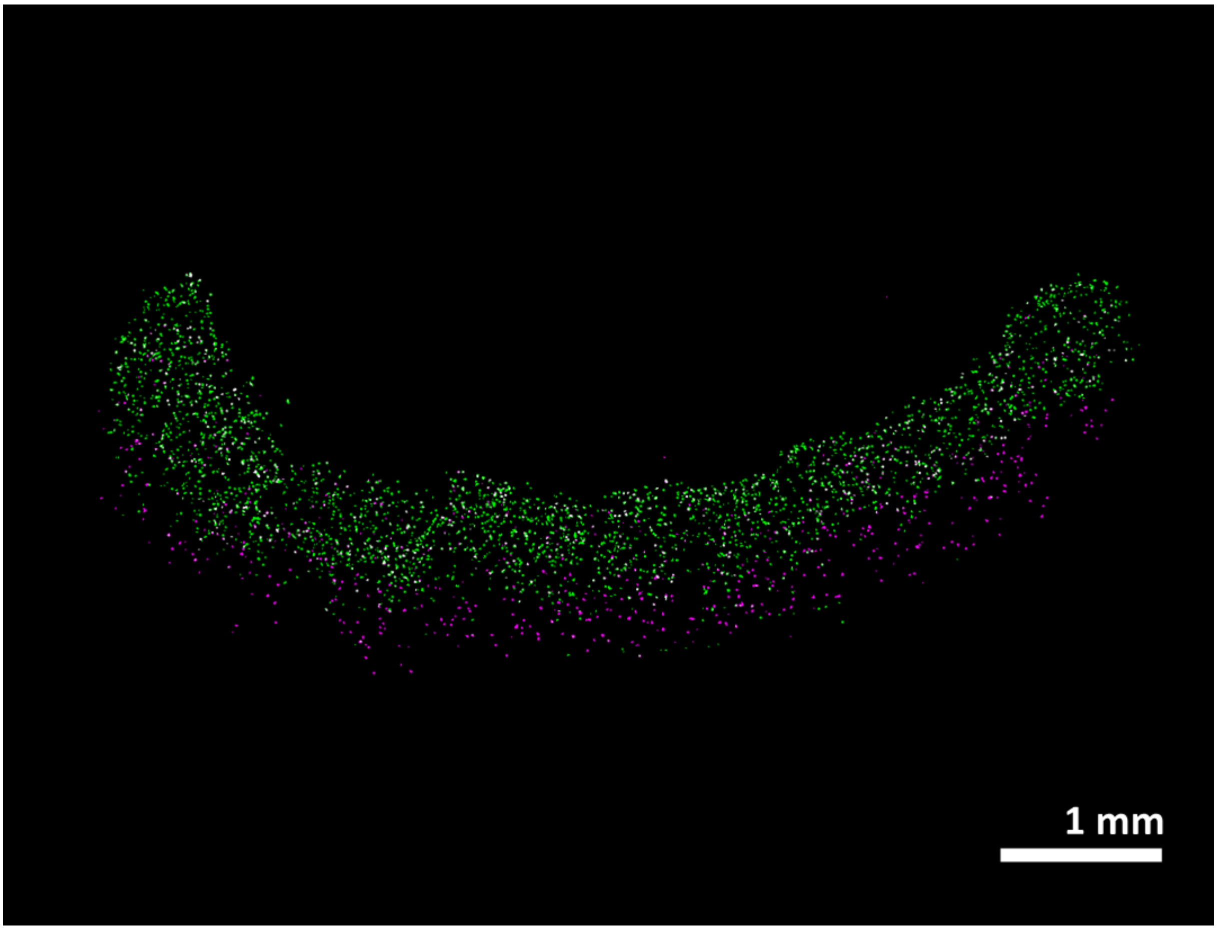

Figure S5. Multilayered 4D bioprinted scaffold. Confocal image displayed in Figure 8h showing the cells from the top layer labelled in green and the bottom layer, in purple.

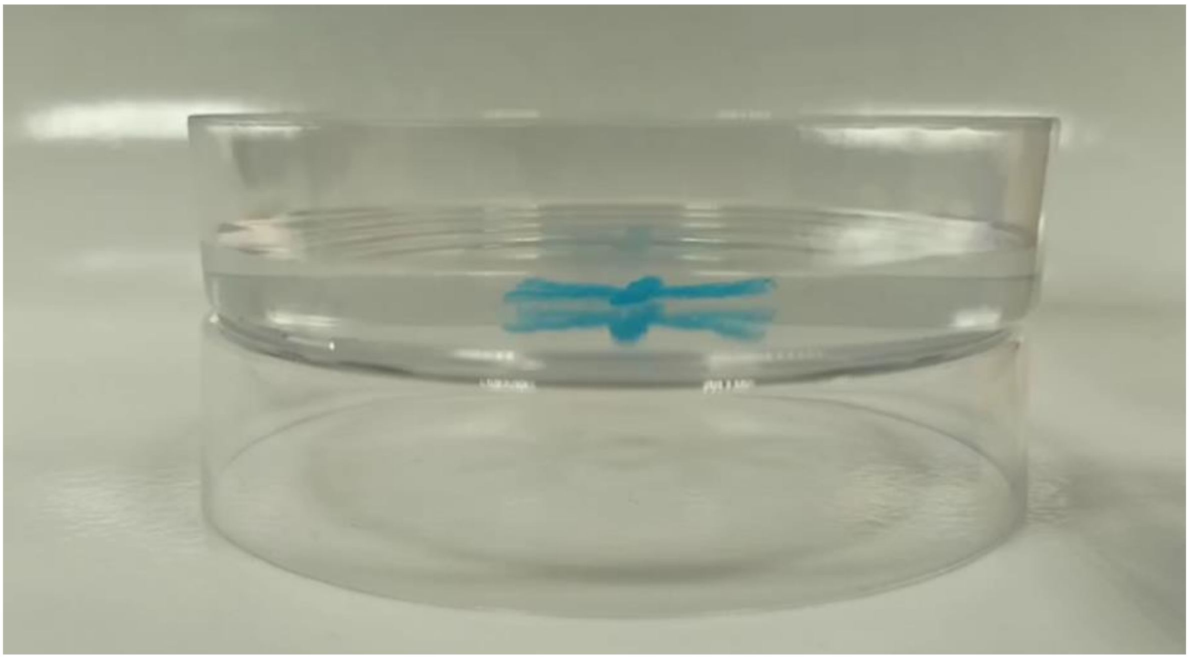

Video S1. Flower-shaped scaffold self-bending upon triggering with swelling solution. Go to video.
